# Supplementary material for: Hema-seq reveals genomic aberrations in a rare simultaneous occurrence of hematological malignancies
Source: Cell Rep Methods. 2023 Oct 17;3(10):100617. doi: 10.1016/j.crmeth.2023.100617 (PMC10626221; doi:10.1016/j.crmeth.2023.100617)
Supplement: Document S1. Figures S1–S5 and Tables S1 and S2 [file mmc1.pdf]

**Cell Reports Methods, Volume *3***

## Supplemental information

# Hema-seq reveals genomic aberrations in a rare simultaneous occurrence of hematological malignancies

**Dajeong Jeong, Amos C. Lee, Kyoungseob Shin, Jinhyun Kim, Myoung Hee Ham, Changhee Lee, Sumin Lee, Ahyoun Choi, Taehoon Ryu, Okju Kim, Yushin Jung, Sunghoon Kwon, and Dong Soon Lee**

Supplementary figures

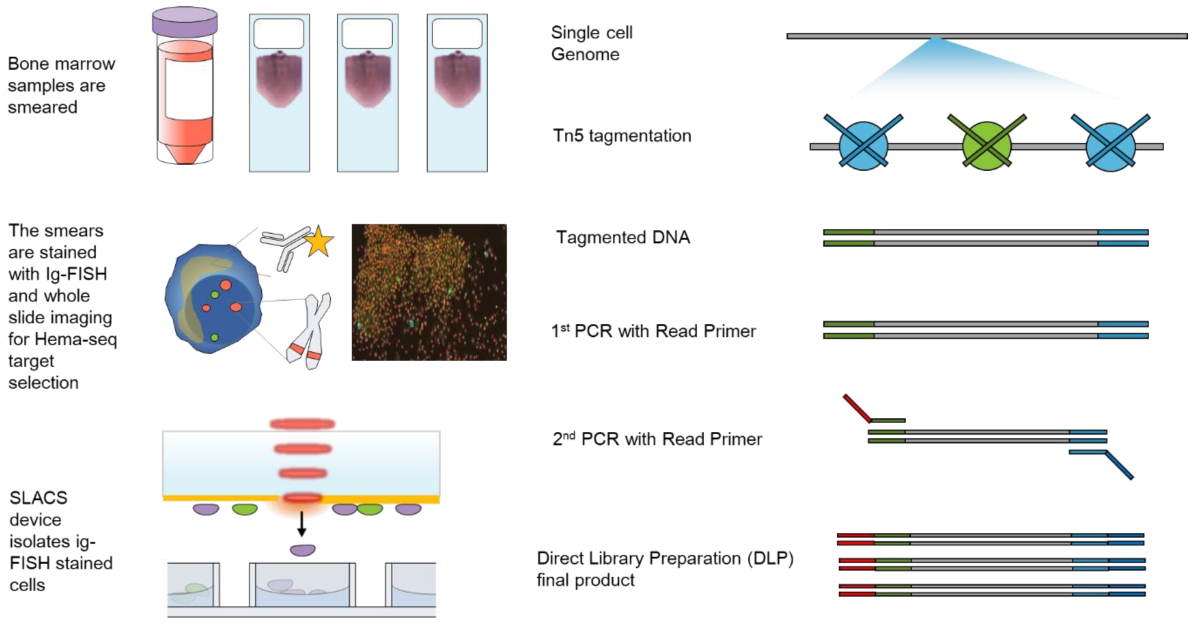


**Figure S1. Hema-seq isolates cytoplasmic-Ig FISH stained cells with SLACS and Direct Library Preparation is performed to amplify genome, related to Figure 1.**

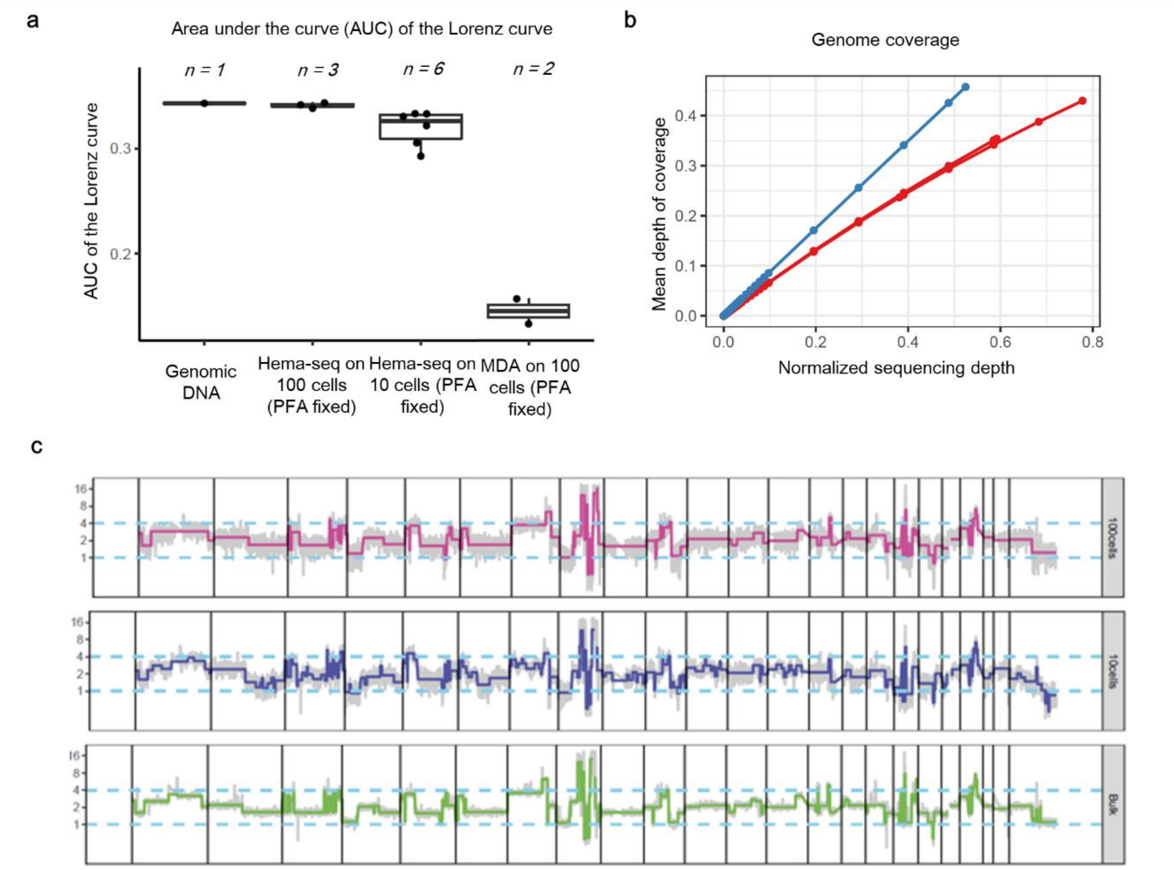


**Figure S2: Hema-seq is validated using the cell line, related to Figure 1.** (**a**) Area under the curve of the Lorenz curve for different methodologies for low-input whole genome sequencing and cell numbers. MDA is an abbreviation for multiple displacement amplification, which is the most widely used whole genome amplification method. (**b**) Genome coverage of the Hema-seq (red) compared to bulk (blue). (**c**) Copy number analysis for comparison between Hema-seq prepared samples (violet and blue) and bulk population (green).

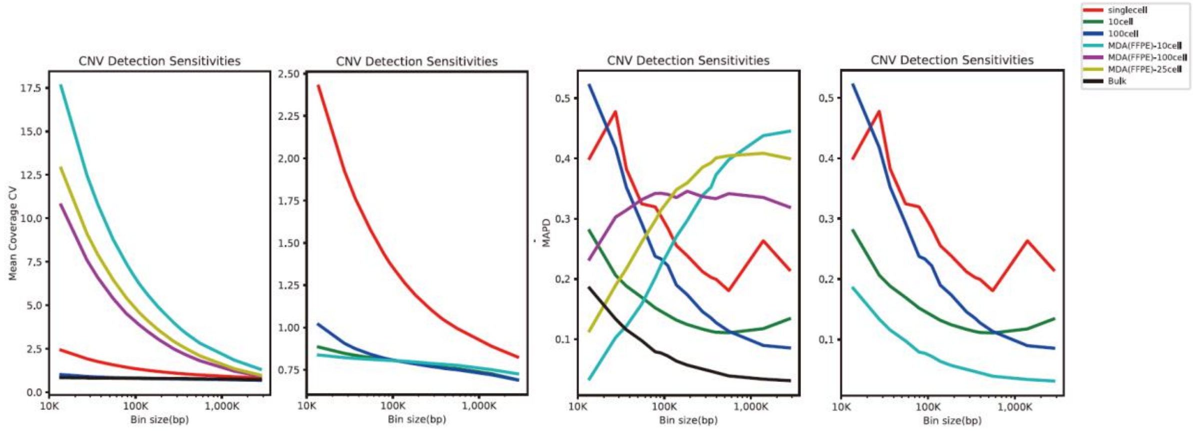


**Figure S3: CNV detection sensitivities of Hema-seq is compared with MDA protocol by mean coverage CV & MAPD of increasing bin size, related to Figure 1.** Hema-seq protocol is applied to single cell(n=9), 10 cells(n=6), 100 cells(n=3) and for comparison MDA protocol is applied to 10 cells(n=2), 25 cells(n=2), 100 cells(n=1) which is performed at FFPE sample. Reference is the bulk sequencing data.

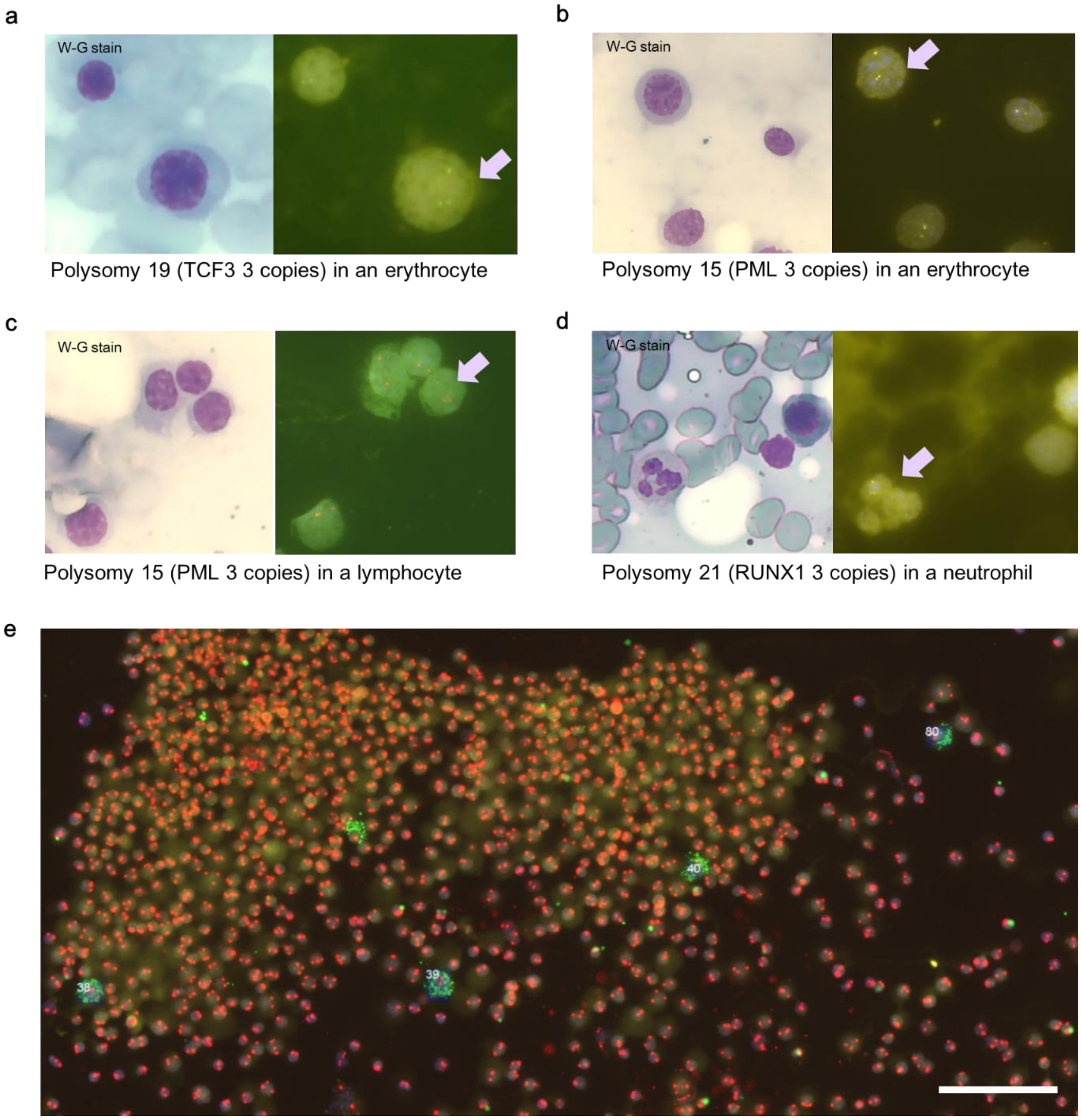


**Figure S4. Wright-Giemsa staining, direct BM smear FISH and cytoplasmic-Ig FISH results of the patient analysed related to Figure 2.** Wright-Giemsa (W-G) staining and direct BM smear FISH results show polysomy 19 (TCF3 3 copies) in an erythrocyte (**a**), polysomy 15 (PML 3 copies) in an erythrocyte (**b**), polysomy 15 (PML 3 copies) in a lymphocyte (**c**), and polysomy 21 (RUNX1 3 copies) in a neutrophil (**d**). (**e**) Cytoplasmic Ig-FISH results and markings for Hema-seq for selective isolation of different cell types. Scale bar, 100 μm

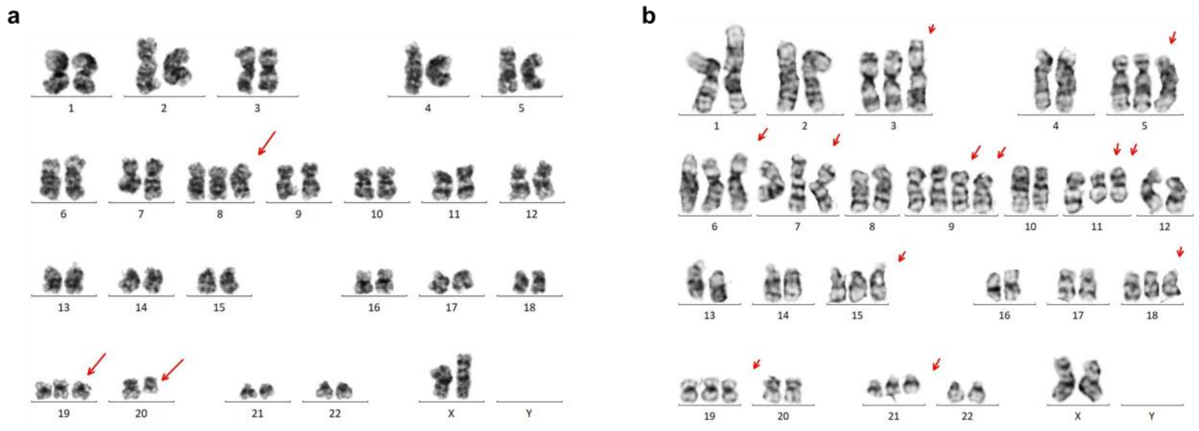

**Figure S5. Karyograms of the analysed person is used for initial diagnosis related to Figure 2**. Clone 1 (48,XX, +8,+19,del(20q)) representing leukemia clone (a) and clone 2 (57,XX, +3, +5,+6,+7,+9,+9,+11,+15,+18,+19,+21) suggestive of plasma cell myeloma clone (b), which were confirmed by interphase FISH and cytoplasmic-Ig FISH. Cytogenetic changes are labelled using red arrows.

**Supplementary Table**

**Table S1.** Target sequencing panel is used to find the single nucleotide variant in the clone, related to Figure 4, Figure 5, Table 1.

*ABCA7, ABCB7, ABCC1, ABL1, ABRAXAS1, ACD, ACKR1, ACTB, ACVR2B, ACYP2, ADA, AK2, AKAP13, AKAP9, AKT1, AKTIP, ALK, ALMS1, ALPK2, AMER1, ANKRD24, ANKRD26, AP3B1, APC, ARHGAP26, ARID1A, ARID1B, ARID2, ARID3A, ARID4B, ASXL1, ASXL2, ASXL3, ATM, ATR, ATRX, B2M, BAP1, BARD1, BAX, BCAS3, BCL2L1, BCL10, BCL11B, BCL2, BCL2L11, BCL6, BCL7A, BCL9, BCOR, BCORL1, BCR, BICD1, BIRC3, BLM, BLNK, BLOC1S6, BMPR1A, BRAF, BRCA1, BRCA2, BRCC3, BRD2, BRD4, BRD7, BRINP3, BRIP1, BRPF1, BTG1, BTG2, BTG3, BTK, BTLA, BUB1, BUB1B, CACNA1E, CALR, CARD11, CARD6, CASP10, CASP8, CBL, CBLB, CBLC, CBX5, CBX7, CCDC80, CCND1, CCND3, CD200, CD27, CD36, CD3D, CD3E, CD40LG, CD58, CD70, CD79A, CD79B, CDC73, CDH1, CDH23, CDK12, CDK4, CDKN1B, CDKN1C, CDKN2A, CDKN2B, CDKN3, CEBPA, CELSR2, CEP164, CHD1, CHD2, CHD8, CHEK2, CHRNA3, CHRNA5, CHRNB4, CIAO1, CIAO2B, CIAO3, CIC, CIITA, CLPTM1L, CMYA5, CNOT1, COIL, COL4A2, COL6A3, CPNE3, CREBBP, CRLF2, CSF1R, CSF3R, CSTF2T, CTC1, CTCF, CTNNB1, CTSS, CUL9, CUX1, CXCL12, CXCR4, CYLD, DAP3, DAXX, DCAF4, DCLK1, DCLRE1B, DCLRE1C, DDB2, DDX1, DDX11, DDX23, DDX3X, DDX41, DDX54, DHX29, DHX32, DHX58, DICER1, DIS3, DIS3L2, DKC1, DKK2, DLEU1, DLEU2, DMD, DNAH9, DNM2, DNMT1, DNMT3A, DNMT3B, DST, DYRK4, EBF1, ECT2L, EED, EEF1E1, EGFR, EGR2, ELANE, EP300, EPCAM, EPHA2, EPHA3, EPHA7, ERBB2, ERCC2, ERCC3, ERCC4, ERCC5, ERCC6, ETS1, ETV3, ETV6, EXT1, EXT2, EZH2, FAH, FANCA, FANCB, FANCC, FANCD2, FANCE, FANCF, FANCG, FANCI, FANCL, FANCM, FAS, FASLG, FAT4, FBXO11, FBXW7, FGFR2, FGFR3, FH, FLCN, FLG, FLT3, FOXL2, FOXN1, FOXO1, FOXP3, FUBP1, FYB1, G6PC3, G6PD, GAR1, GATA1, GATA2, GATA3, GEMIN2, GFI1, GLI1, GNA11, GNA13, GNAQ, GNAS, GNB1, GPC3, GPC4, GSTP1, HAX1, HCK, HDAC2, HDAC3, HDAC7, HEATR1, HIST1H1E, HNF1A, HNRNPK, HMBOX1, HOXB13, HRAS, HUWE1, IDH1, IDH2, IKZF1, IKZF2, IKZF3, IL2RG, IL7R, IRAK1, IRAK4, IRF1, IRF4, IRF8, ITK, ITPKB, JAK1, JAK2, JAK3, JMJD1C, KDM2B, KDM3B, KDM4C, KDM5C, KDM6A, KDM6B, KDR, KIAA0355, KIF20B, KIT, KLF6, KLHL6, KLK3, KMT2A, KMT2B, KMT2C, KMT2D, KRAS, L2HGDH, LAMB4, LAMTOR2, LEF1, LIG4, LMO2, LRP1B, LRRK1, LSP1, LUC7L2, LYST, MAD1L1, MAGT1, MALT1, MAP2K1, MAP2K2, MAP2K4, MAP3K1, MAP3K14, MAP4K1, MAPK1, MAX, MDM2, MED12, MEF2B, MEF2C, MEN1, MET, METTL3, MIR155, MKI67, MLH1, MMS19, MPDZ, MPHOSPH6, MPL, MRE11, MSH2, MSH6, MSMB, MST1R, MTA2, MTOR, MUC16, MUTYH, MXRA5, MYB, MYC, MYCN, MYD88, MYLK2, MYO3A, NAF1, NBN, NCF4, NF1, NF2, NFE2L2, NFKB2, NFKBIA, NFKBIE, NHEJ1, NHP2, NKX2-1, NOP10, NOTCH1, NOTCH2, NOTCH3, NPM1, NR3C1, NRAS, NRK, NSD1, NTRK1, NUP214, OBSCN, OR6K3, ORAI1, P2RY8, PALB2, PALLD, PARD3, PARP1, PARP2, PASD1, PASK, PAX5, PBRM1, PCLO, PDGFC, PDGFRA, PDGFRB, PDS5B, PDSS2, PHF6, PHLPP1, PHOX2B, PIF1, PIGA, PIGT, PIK3C3, PIK3CA, PIK3R1, PIM1, PINX1, PKD1L2, PLCG2, PLEKHG5, PLRG1, PML, PMS1, PMS2, PNP, POLG, POLH, POLR2A, POSTN, POT1, POU2F2, PPP2R1A, PRDM1, PRDM16, PRDM9, PRF1, PRKAR1A, PRKCG, PRKD3, PRKDC, PRPF3, PRPF40B, PRPF8, PRSS1, PTCH1, PTEN, PTPN11, PTPN14, PTPRC, PTPRT, PWWP3A, RAB27A, RAC2, RAD21, RAD50, RAD51, RAD51AP1, RAD51B, RAD51C, RAD51D, RAD54L, RAF1, RAG1, RAG2, RAPGEF1, RARA, RB1, RBBP4, RBMX,*

*RECQL, RECQL4, RECQL5, REL, RELN, RET, RFTN1, RFX7, RFXAP, RING1, RIPK1, RIT1, RMRP, RNF213, RPL11, RPL19, RPL27, RPL35A, RPL5, RPN1, RPS10, RPS14, RPS17, RPS19, RPS24, RPS26, RPS27, RPS6KA6, RPS7, RTEL1, RUNX1, SAMD9, SAMD9L, SAMHD1, SAP130, SBDS, SCML2, SCRIB, SDHA, SDHAF2, SDHB, SDHC, SDHD, SENP6, SETBP1, SETD2, SF1, SF3A1, SF3B1, SGK1, SH2B3, SH2D1A, SHOC2, STK11, SLC37A4, SLC7A7, SLITRK6, SLX4, SMAD1, SMAD4, SMAD7, SMARCA2, SMARCA4, SMARCB1, SMARCD2, SMARCE1, SMC1A, SMC3, SMC5, SMG1, SMO, SNRNP200, SOCS1, SOS1, SPEN, SPINK1, SRP72, SRRM2, SRSF2, SRSF6, SRSF8, STAG2, STAT3, STAT5B, STAT6, STIM1, STK4, STK11, STK32A, STK33, STK36, STN1, STRIP2, STX11, STXBP2, SUDS3, SUFU, SUMO2, SUPT5H, SUZ12, SYK, SYNE1, TAF1, TAL1, TAZ, TBL1XR1, TBX1, TCF12, TCF4, TEN1, TENT4B, TENT5C, TEP1, TERC, TERF1, TERF2, TERF2IP, TERT, TET1, TET2, TGFB2, TGM6, TGM7, THPO, THRB, TINF2, TMEM127, TMEM30A, TNFAIP3, TNFRSF14, TNFSF9, TNKS, TNKS1BP1, TNKS2, TOX3, TP53, TRA2B, TRAF3, TRIM37, TRIO, TSC1, TSC2, TSHR, TTBK1, TTC27, TTN, TYK2, TYW1, U2AF1, U2AF1L4, U2AF2, UBA3, UBE2A, UGGT1, ULK4, UNC13B, UNC13D, UNC5C, UNC5D, USB1, VHL, VPS13A, VPS13B, VPS45, WAC, WAPAL, WAS, WEE1, WIPF1, WNK3, WNK4, WRAP53, WRN, WT1, XIAP, XPA, XPC, XPO1, XRCC2, XRCC6, ZAP70, ZBTB33, ZBTB7B, ZFHX3, ZMYM3, ZNF208, ZNF311, ZNF608, ZNF676, ZNF708, ZRSR2*

**Table S2**. Results of direct BM smear FISH on neutrophil, erythrocytes, lymphocytes, neutrophil and megakaryocytes, related to Figure 2.

| Direct BM smear FISH | Neutrophil | Erythrocyte | Lymphocyte | Megakaryocytes |
|---|---|---|---|---|
| Polysomy 19 (TCF3 3 copies) | 0/13 (0.0%) | 4/68 (5.9%) | 0/11 (0.0%) | N/A |
| Polysomy 8 (CEP8 3 copies) | 0/7 (0.0%) | 0/188 (0.0%) | 0/52 (0.0%) | 0/0 (0.0%) |
| Polysomy 11 (MLL 3 copies) | 0/6 (0.0%) | 1/17 (5.9%) | 0/16 (0.0%) | N/A |
| Polysomy 15 (PML 3 copies) | 0/38 (0.0%) | 5/209 (2.4%) | 4/113 (3.5%) | 0/0 (0.0%) |
| Polysomy 21 (RUNX1 3 copies) | 1/3 (33.3%) | 0/5 (0.0%) | 0/10 (0.0%) | 0/0 (0.0%) |
