# Supplementary material for: Hema-seq reveals genomic aberrations in a rare simultaneous occurrence of hematological malignancies
Source: Cell Rep Methods. 2023 Oct 17;3(10):100617. doi: 10.1016/j.crmeth.2023.100617 (PMC10626221; doi:10.1016/j.crmeth.2023.100617)
Supplement: Document S2. Article plus supplemental information [file mmc2.pdf]

Cell Reports
Methods

Report

# Hema-seq reveals genomic aberrations in a rare simultaneous occurrence of hematological malignancies

## Graphical abstract

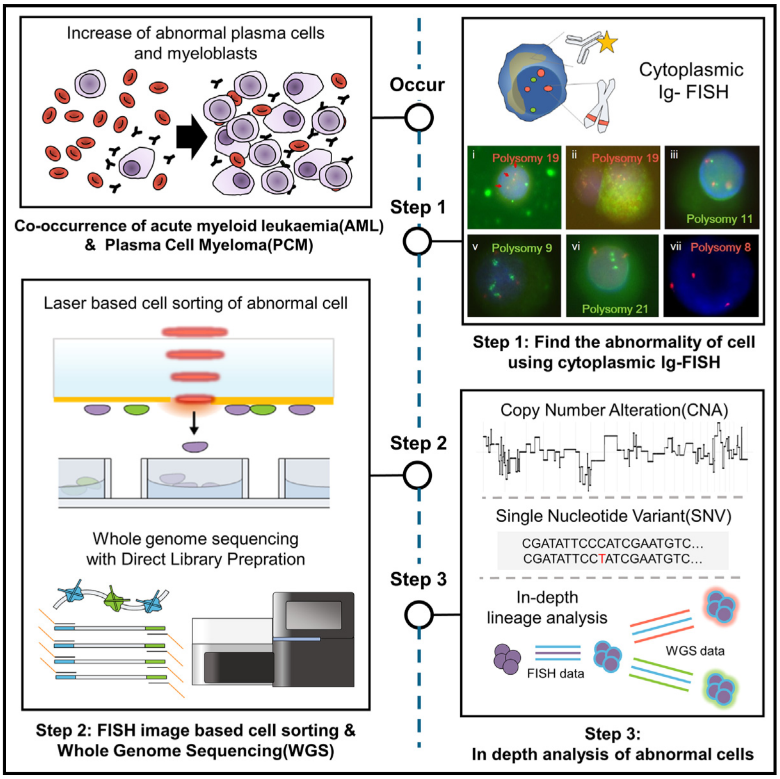


## Highlights

- Hema-seq integrates genomics and cytopathology
- A rare convergence of myeloma and leukemia is explored with Hema-seq
- Hema-seq reveals clonal changes in hematopoietic lineages
- Hema-seq can enable a deeper genomic analysis of archival samples

## Authors

Dajeong Jeong, Amos C. Lee, Kyoungseob Shin, ..., Yushin Jung, Sunghoon Kwon, Dong Soon Lee

## Correspondence

skwon@snu.ac.kr (S.K.),
soonlee@snu.ac.kr (D.S.L.)

## In brief

Jeong et al. present Hema-seq, a method for combining cytopathological and genomic profiling to reconstruct blood tumor lineages. They analyze a complex case of simultaneous myeloma and AML, identifying clonal shifts within the hematopoietic lineage. Hema-seq is compatible with archival samples and offers fresh opportunities to understand complex hematological conditions.

Jeong et al., 2023, Cell Reports Methods *3*, 100617
October 23, 2023 © 2023 The Authors.
https://doi.org/10.1016/j.crmeth.2023.100617

CellPress

Cell Reports Methods

CellPress
OPEN ACCESS

Report

# Hema-seq reveals genomic aberrations in a rare simultaneous occurrence of hematological malignancies

Dajeong Jeong,[1,9] Amos C. Lee,[2,3,9] Kyoungseob Shin,[4,9] Jinhyun Kim,[4] Myoung Hee Ham,[1] Changhee Lee,[4] Sumin Lee,[3,4] Ahyoun Choi,[5] Taehoon Ryu,[6] Okju Kim,[6] Yushin Jung,[6] Sunghoon Kwon,[2,4,5,7,8,10,*] and Dong Soon Lee[1,8,*]

[1]Department of Laboratory Medicine, Seoul National University Hospital, Seoul 03080, Republic of Korea
[2]Bio-MAX Institute, Seoul National University, Seoul 08826, Republic of Korea
[3]Meteor Biotech Co., Ltd., Seoul 08826, Republic of Korea
[4]Department of Electrical and Computer Engineering, Seoul National University, Seoul 08826, Republic of Korea
[5]Interdisciplinary Program in Bioengineering, Seoul National University, Seoul 08826, Republic of Korea
[6]ATG Lifetech, Inc., Seoul 08507, Republic of Korea
[7]Institutes of Entrepreneurial BioConvergence, Seoul National University, Seoul 08826, Republic of Korea
[8]Cancer Research Institute, Seoul National University College of Medicine, Seoul 03080, Republic of Korea
[9]These authors contributed equally
[10]Lead contact
*Correspondence: skwon@snu.ac.kr (S.K.), soonlee@snu.ac.kr (D.S.L.)
https://doi.org/10.1016/j.crmeth.2023.100617

**MOTIVATION** Hematological malignancies, with their intricate molecular landscapes, present significant challenges in the diagnostic and therapeutic realms. The co-existence of neoplastic and normal cells within these malignancies further adds to this complexity. Traditional cytogenetic methods, while invaluable, cannot resolve the full spectrum of genomic aberrations, especially in rare simultaneous occurrences. We therefore sought to bring the greater degree of genomic resolution afforded by whole-genome sequencing to samples that have been processed and preserved through cytogenetic methods and to link sequencing profiles to selected cell populations for higher-resolution clonal reconstruction and analysis. Such an approach not only holds promise in unveiling the mysteries of clonal evolution but also in guiding tailored therapeutic strategies for enhanced patient outcomes. In short, our work with "Hema-seq" is driven by the motivation to bridge existing gaps and offer a panoramic view of the genomic intricacies within hematopoietic lineage cells.

## SUMMARY

Co-occurrence of multiple myeloma and acute myelogenous leukemia is rare, with both malignancies often tracing back to multipotent hematopoietic stem cells. Cytogenetic techniques are the established baseline for diagnosis and characterization of complex hematological malignancies. In this study, we develop a workflow called Hema-seq to delineate clonal changes across various hematopoietic lineages through the integration of whole-genome sequencing, copy-number variations, cell morphology, and cytogenetic aberrations. In Hema-seq, cells are selected from Wright-stained slides and fluorescent probe-stained slides for sequencing. This technique therefore enables direct linking of whole-genome sequences to cytogenetic profiles. Through this method, we mapped sequential clonal alterations within the hematopoietic lineage, identifying critical shifts leading to myeloma and acute myeloid leukemia (AML) cell formations. By synthesizing data from each cell lineage, we provided insights into the hematopoietic tree's clonal evolution. Overall, this study highlights Hema-seq's capability in deciphering genomic heterogeneity in complex hematological malignancies, which can enable better diagnosis and treatment strategies.

## INTRODUCTION

In hematological malignancies, the high genetic heterogeneity limits the investigation of the hematopoietic lineage of abnormal cells. Especially, profiling genetic abnormalities in bulk specimen of bone marrow (BM) aspirates gives mixed genetic information of different cell lineages. Although rare, understanding the origins of these neoplastic cells is of utmost importance in diagnosing and


This is an open access article under the CC BY license (http://creativecommons.org/licenses/by/4.0/).

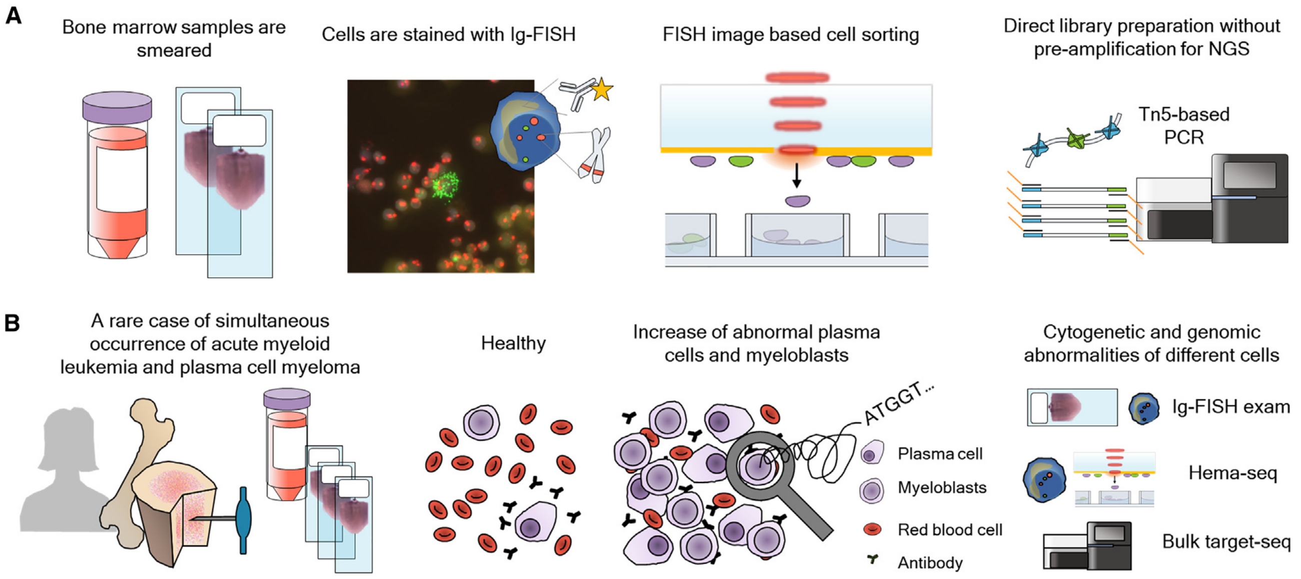


**Figure 1. Whole-genome sequencing of cytoplasmic-Ig FISH-stained cells of interest and its application in a rare case of simultaneous AML and PCM**

(A) Hema-seq isolates cytoplasmic-Ig FISH-stained cells of interest and adopts direct library preparation without pre-amplification for whole-genome sequencing.

(B) The simultaneous occurrence of acute myeloid leukemia (AML) and plasma cell myeloma (PCM) was comprehensively analyzed using cytoplasmic-Ig FISH, Hema-seq, and bulk target sequencing.

treating patients with rare disease, who are often neglected.[1] One example is the rare simultaneous occurrence of acute myeloid leukemia (AML) and plasma cell myeloma (PCM). A few case reports investigating the cytogenetic characteristics and/or the origin of progenitors of the two types of malignant clones have been published. Wang et al. performed immunomagnetic separation of plasma cells (PCs), which were found to harbor amplifications of *RB1*, *TP53*, and *CDKN2C*.[2,3] Previous work suggested common leukemic progenitors of myeloblasts (MBs) and PCs using flow cytometry, Southern blot, and methylcellulose assay.[4] However, in highly heterogeneous hematologic malignancies, genomic abnormalities must be identified to understand hematopoiesis in different cell subpopulations. PC subpopulations in a pool of hematopoietic cells can be identified using cytoplasmic immunoglobulin fluorescence *in situ* hybridization (Ig-FISH). The cytoplasmic Ig-FISH procedure includes staining of cytoplasm with Ig fluorescent antibody and subsequent staining with target FISH probe. Using cytoplasmic Ig-FISH, we could observe cytogenetic abnormalities of PC populations that express Ig. To identify MB populations, Wright-Giemsa staining is used to identify MBs, and interphase FISH is performed on top of the Wright-Giemsa-identified MBs (direct BM smear FISH). In other words, interphase FISH techniques including cytoplasmic Ig-FISH and direct BM smear FISH or Wright-Giemsa staining serve to identify and categorize cell populations that cannot be sorted with conventional flow cytometry techniques. The simultaneous occurrence of AML and PCM is not only poorly documented, but the high heterogeneity of cell subpopulations has not been resolved with whole-genome sequencing. Despite the need to analyze whole genomes of cytogenetically categorized lineage cells, whole-genome profiling of cytogenetically typed subpopulations remains technically challenging due to two reasons. First, categorizing cytogenetically typed cells and sorting them accordingly requires highly sensitive single-cell sorting technology that can delineate fluorescence signals from the interphase FISH-stained cells. Second, the paraformaldehyde (PFA) fixation and FISH inhibit enzymes used in widely used whole-genome amplification technologies such as multiple displacement amplification[5] or multiple annealing and looping-based amplification cycles.[6]

In this study, we report a case of simultaneous occurrence of AML and PCM and reveal cytogenetic and cryptic whole-genome profiles of different cells that are cataloged by cell morphology with Wright-Giemsa-stained, direct BM smear FISH-stained, and cytoplasmic-Ig FISH-stained slides. We developed Hema-seq by advancing our previously developed PHLI-seq,[7] which uses multiple displacement amplification that is incompatible with PFA-fixed cells. Instead, Hema-seq utilizes Tn5 transposase-based library preparation (direct library preparation [DLP]) without pre-amplification[8] in PFA-fixed direct BM smear FISH-stained, cytoplasmic Ig-FISH-stained, or Wright-Giemsa-stained cells (Figures 1A and S1–S3). The main advancement from PHLI-seq is that we use not only DLP for whole-genome amplification but also reverse cross-linking using proteinase K. Hema-seq is a gold-standard-friendly technique that is compatible with conventional protocols for direct BM smear FISH, cytoplasmic Ig-FISH, and Wright-Giemsa staining (Figure S4). In addition to the developed Hema-seq, cytoplasmic Ig-FISH, direct BM smear FISH, and bulk targeted sequencing were performed (Figure 1B). The interphase FISH exams and bulk targeted sequencing not

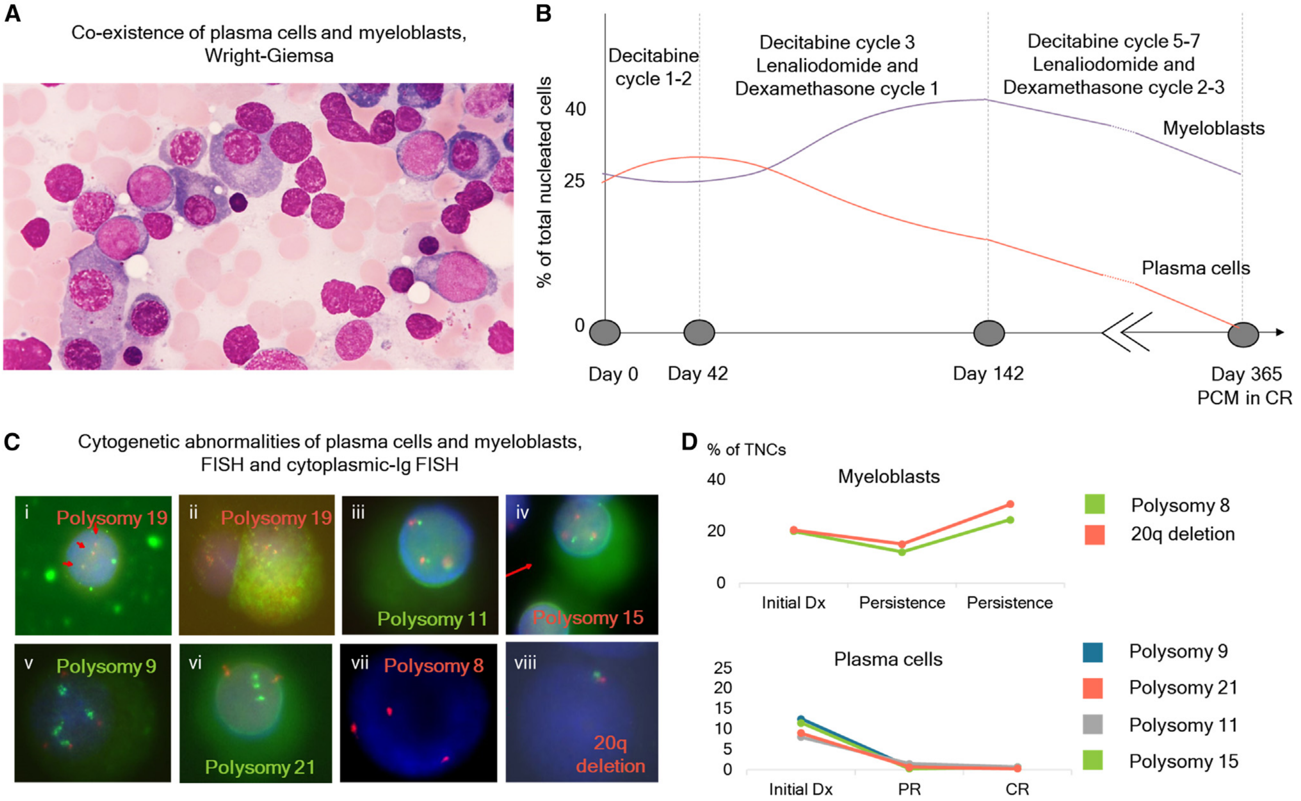


**Figure 2. Characterization of the patient with simultaneous occurrence of AML and PCM**

(A) Co-existence of plasma cells and myeloblasts was confirmed by Wright-Giemsa staining.

(B) Patient treatment history.

(C) Cytogenetic abnormalities of plasma cells and myeloblasts were examined using FISH and cytoplasmic Ig-FISH. The *TCF3* gene probe was used in (i) and (ii), MLL in (iii), PML in (iv), CDKN2A for (v), RUNX1 for (vi), CEP8 for (vii), and 20q for (viii).

(D) Percentage of changes in myeloblasts and plasma cells examined using cytoplasmic-Ig FISH.

only validated the Hema-seq data but also provided complementary information. Hema-seq reveals the genomic aberrations of different cells cataloged with cell staining at the whole-genome level. Cytoplasmic-Ig and direct BM smear FISH examination provides the quantity of various cell types, while the bulk targeted sequencing data provide the change in mutation burden as the treatment progresses. Therefore, combining direct BM smear FISH, cytoplasmic-Ig FISH, and bulk targeted sequencing with Hema-seq provides insights into how the various cell populations with different genomic aberrations change as the treatment progresses. Utilizing this advantage, we investigated a rare case of the simultaneous occurrence of AML and PCM. For validation, the copy-number alteration results from Hema-seq were compared with those from interphase FISH, cytoplasmic-Ig FISH, and direct BM smear FISH examination, and the single-nucleotide variation results from Hema-seq were compared with those from the bulk targeted sequencing.

## RESULTS

A 69-year-old woman underwent a regular check-up in which pancytopenia (hemoglobin, 10.6 g/dL; absolute neutrophil count, 0.45 × $10^9$/L; platelet count, 73 × $10^9$/L) was noted. She had hypertension, which was controlled with medication. BM examination was performed under suspicion of hematologic malignancy. MBs and PCs accounted for 24.4% and 25.0% of the total nucleated cells (TNCs), respectively, on BM aspiration (Figure 2A). Based on BM morphology, immunohistochemical staining, flow cytometry, and serum M-protein levels, she was diagnosed with AML with myelodysplasia-related changes (AML-MRCs) and PCM (Figure 2A). Secondary myeloid neoplasms were ruled out because the patient had no history of prior cytotoxic therapy or exposure to radiation.

Initially, the patient was treated with decitabine (cycles 1–2). After 42 days, a follow-up BM study revealed no significant changes in the burden of leukemic blasts and clonal PCs (24.6% and 28.8% of TNCs, respectively) (Figure 2B). Lenalidomide and dexamethasone therapy (cycle 1) was initiated with decitabine (cycles 3–4). After 142 days the initial diagnosis, the third BM study showed persistent leukemic blasts (39.4% of TNCs) and decreased neoplastic PCs (15.8% of TNCs), which led to the achievement of partial remission of PCM. Additionally, she received decitabine (cycles 5–7) and lenalidomide and dexamethasone therapy (cycles 2–3). Approximately 1 year after the initial diagnosis, the fourth BM study showed persistent residual

**Table 1. Laboratory, cytogenetic, and molecular characteristics of five consecutive BM specimens from the patient with concurrent AML and PCM**

| Specimen number | 1 | 2 | 3 | 4 |
|---|---|---|---|---|
| Days after initial Dx | 0 | 42 | 142 | 364 |
| BM | | | | |
| Blast count, % | 24.4 | 24.6 | 39.4 | 26.4 |
| PC count, % | 25.0 | 28.8 | 15.8 | 2.0 |
| Protein immunochemistry | | | | |
| Serum K/L | 0.16 | 0.09 | 0.76 | NT |
| Serum M protein, g/dL | 1.02 | 1.07 | 0.18 | 0.00 |
| Karyotype | 47,XX,+8,del(20)(q11.2)[7]/ 48,idem,+19[8]/46,XX[9] | 47,XX,+8,del(20)(q11.2)[1]/ 48,idem,+19[9]/55–57,XX, +3,+5,+6,+7,+9,+9,+11, ?del(11)(q14q23)x2,+15,+18, +19,+21 [cp3]/46,XX[7] | 47,XX,+8,del(20)(q11.2)[1]/ 48,idem,+19[3]/46,XX[17] | 47,XX,+8,del(20)(q11.2)[1]/ 48,sl,+19[6]/94,slx2[1]/ 46,XX[12] |
| FISH,[a] cytoplasmic-Ig FISH | | | | |
| Polysomy 19 (*TCF3* 3 copies) | | | | |
| % in TNCs | 22.0 (44/200) | NT | 11.5 (23/200) | 20.0 (40/200) |
| Non-PC:PC | 0.9 (21:23) | – | 7.0 (20:3) | 12.0 (37:3) |
| % in PCs | 95.0 (19/20) | NT | 30.0 (3/10) | 30.0 (3/10) |
| Polysomy 9 (*CDKN2A* tetrasomy) | | | | |
| % in TNCs | 12.5 (25/200) | NT | 0.7 (2/300)[b] | 0.7 (2/300)[b] |
| % in non-PCs | ND | NT | ND | ND |
| % in PCs | 100 (20/20) | NT | 35 (7/20) | 0.0 (0/20) |
| Polysomy 21 (*RUNX1* 3 copies) | | | | |
| % in TNCs | 11.5 (23/200) | NT | 0.3 (1/300) | 0.7 (2/300) |
| % in non-PCs | ND | NT | ND | ND |
| % in PCs | 82.0 (41/50) | NT | 20.0 (2/10) | 20.0 (1/5) |
| Polysomy 11 (*MLL* 3 copies) | | | | |
| % in TNCs | 8.0 (16/200) | NT | 1.5 (3/200) | 0.7 (2/300) |
| % in non-PCs | ND | NT | ND | ND |
| % in PCs | 90.0 (18/20) | NT | 30.0 (3/10) | 20.0 (2/10) |
| Polysomy 15 (*PML* 3 copies) | | | | |
| % in TNCs | 9.0 (18/200) | NT | 0.7 (2/300) | 0.3 (1/300) |
| % in non-PCs | ND | NT | ND | ND |
| % in PCs | 100.0 (20/20) | NT | 20.0 (2/10) | 28.5 (2/7) |
| Polysomy 8 (CEP8 3 copies) | | | | |
| % in TNCs | 20.0 (40/200) | NT | 12.0 (24/200) | 24.5 (49/200) |
| % in PCs | 0.0 (0/20) | NT | 0.0 (0/20) | 0.0 (0/8) |
| 20q deletion | | | | |
| % in TNCs | 20.5 (41/200) | NT | 15.0 (30/200) | 30.5 (61/200) |
| % in PCs | 0.0 (0/20) | NT | 0.0 (0/20) | 0.0 (0/20) |
| Target sequencing | | | | |
| Somatic variants, VAF (%) | | | | |
| *DDX41* (c.679A>G) | 3.6 | 4.4 | 9.5 | 12.4 |
| *BAX* (c.280C>T) | 8.4 | 9.5 | 8.0 | 16.8 |
| *ATM* (c.1262C>A) | 4.7 | 2.6 | 2.5 | ND |
| *ZNF676* (c.1564G>T) | 4.2 | ND | ND | ND |
| *PRPF3* (c.11C>A) | 1.2 | ND | ND | ND |
| *GPC3* (c.352G>T) | 2.2 | ND | ND | 1.7 |
| *GPC3* (c.355G>T) | 1.9 | ND | ND | ND |

(*Continued on next page*)

**Table 1. *Continued***

| Specimen number | 1 | 2 | 3 | 4 |
|---|---|---|---|---|
| *KMT2C* (c.2576G>T) | 13.2 | ND | ND | ND |
| *KDR* (c.409T>G) | 2.2 | ND | ND | ND |
| Tx | N/A | s/p decitabine C1, C2 | s/p decitabine C3–C4 lenalidomide + dexamethasone C1 | s/p decitabine C5–C7 lenalidomide + dexamethasone C2–C3 |
| Tx response | | | | |
| AML-MRC | N/A | persistence | persistence | persistence |
| PCM | N/A | no significant interval change | partial Remission | complete Remission |

AML-MRC, acute myeloid leukemia with myelodysplasia-related changes; BM, bone marrow; C, cycle; Dx, diagnosis; K/L, kappa-to-lambda ratio; N/A, not applicable; ND, not detected; NT, not tested; PCM, plasma cell myeloma; s/p, status post; TNC, total nucleated cell; Tx, treatment; VAF, variant allele frequency.

[a]FISH cutoffs (%) for each abnormality are as follows: TCF3 3 copy (F3), 2.0 (100 cells), 1.5 (200 cells), 1.3 (300 cells); CDKN2A tetrasomy (O4G4), 1.0 (100 cells), 1.0 (200 cells), 1.0 (300 cells); RUNX1 3 copies (O3G2), 1.0 (100 cells), 0.5 (200 cells), 0.3 (300 cells); MLL3 3 copies (F3), 1.0 (100 cells), 0.5 (200 cells), 0.3 (300 cells); PML3 3 copies (O3G2), 0.0 (100 cells), 0.5 (200 cells), 0.3 (300 cells); and CEP 8 trisomy (O3), 2.0 (100 cells), 1.5 (200 cells), 1.3 (300 cells); 20q del (O1G1), 3.0 (100 cells), 2.0 (200 cells), 1.7 (300 cells).

[b]Less than the cutoff value.

MBs (26.4% of TNCs). Meanwhile, the PCM was in complete remission according to the International Myeloma Working Group Response Criteria (Table 1). Giemsa banding karyotyping, cytoplasmic-Ig FISH, direct BM smear FISH, and targeted next-generation sequencing using a gene panel consisting of 642 hematological malignancy and cancer-related genes were performed on serial follow-up BM specimens (Table S1).

Comprehensive analysis using interphase FISH revealed common and/or discrete cytogenetic abnormalities in PCs and MBs. Polysomy 19 (TCF3 three copies) was present in both PCs and MBs, implying that clones with polysomy 19 originated from common progenitor cells. Polysomies 9, 11, 15, and 21 (CDKN2A tetrasomy, MLL3 three copies, PML3 three copies, and RUNX1 three copies) were identified only in cytoplasmic-Ig-positive PCs. Alternatively, polysomy 8 (CEP8 three copies) and 20q deletion were detected only in cytoplasmic-Ig-negative MBs (Figure 2C). PC-specific cytogenetic abnormalities (polysomy 9, 11, 15, and 21) decreased in accordance with the decline of clonal PCs in the BM, while MB-specific abnormalities (polysomy 8 and 20q deletion) persisted during chemotherapy. Meanwhile, the percentage of polysomy 19 out of TNCs, the common cytogenetic abnormality in PCs and MBs, remained high during the follow-up period. The relative proportion of polysomy19 in PCs and MBs showed dynamic changes, depending on the proportion of PCs and MBs. Polysomy 19 was initially predominant in PCs. However, the relative portion in PCs decreased in proportion to the decline of neoplastic PCs, while the relative proportion of MBs increased (Figure 2D).

Meanwhile, in the initial Wright-Giemsa-stained BM aspirate slide, direct BM smear FISH was performed to characterize cytogenetic abnormalities of neutrophils, lymphocytes, erythrocytes, and megakaryocytes. A total of 33% neutrophils revealed polysomy 21, whereas 3.5% lymphocytes harbored polysomy 15. Erythrocytes harbored polysomies 19 and 11 in 5.9% of the cells and polysomy 15 in 2.4% of the cells. Evidence of abnormal cytogenetic aberrations could not be identified in megakaryocytes (Table S2).

In the second follow-up BM, which showed persistent AML and PCM, the FISH- and cytoplasmic-Ig FISH-stained cells underwent Hema-seq. The cells were categorized into 8 different groups: two subgroups of PCs (PCs with polysomy 19 and PCs with polysomy 11), two subgroups of MBs (MBs with three copies of *TCF3* and MBs with 20q deletion), neutrophils, eosinophils, erythrocytes, and megakaryocytes. We sorted the cytoplasmic-Ig FISH-, direct BM smear FISH-, and/or Wright-Giemsa-stained cells with a spatially resolved laser-activated cell sorting (SLACS) device to analyze their copy-number alterations using DLP[8,9] and whole-genome sequencing. We categorized PCs and MBs into two subgroups based on cytoplasmic-Ig FISH results, generating a total of four subgroups of PCs and MBs. Different cell types that were categorized by cytoplasmic-Ig FISH, direct BM smear FISH, or Wright-Giemsa staining were isolated and sequenced using Hema-seq. The results of the copy-number analysis matched those revealed by Giemsa banding (Figure S5), direct BM smear FISH, and cytoplasmic-Ig FISH. Newly detected copy-number alterations by whole-genome sequencing (WGS) were as follows: TP53, chromosome 2, 4q, and X losses in PCs; polysomy 6 in MB, polysomy 16, and chromosome 10 loss in neutrophils and eosinophils; and chromosome 4 loss in erythrocytes.

We also matched the bulk targeted sequencing data to the Hema-seq data for complementation and cross-validation. Bulk targeted sequencing revealed somatic variants as follows: *DDX41*: c.679A<G (Genbank: NM_016222.2) (p.Thr277Ala)NM_016222.2) (p.Thr277Ala)BAX: c.28NM_004324.3) (p.Arg94*); *ATM*: c.1262C>A (Genbank: NM_000051.3) (p.Ser421*); *ZNF676*: c.1564G>T (Genbank: NM_001001411.2): (p.Glu522*); *PRPF3*: c.11C>A (Genbank: NM_004698.2): (p.Ser4*); *GPC3*: c.352G>T (Genbank: NM_001164617.1): (p.Val118Phe); *GPC3*: c.355G>T(Genbank: NM_001164617.1) (p.Val119Phe); *KMT2C*: c.2576G>T (Genbank: XM_005250025.1): (p.Trp859Leu); and *KDR*: c.409T>G (Genbank: NM_002253.2): (p.Tyr137Asp). Mutations in *DDX41* and *BAX* persisted during the follow-up period, whereas the *ATM* mutation disappeared at the time of complete remission of

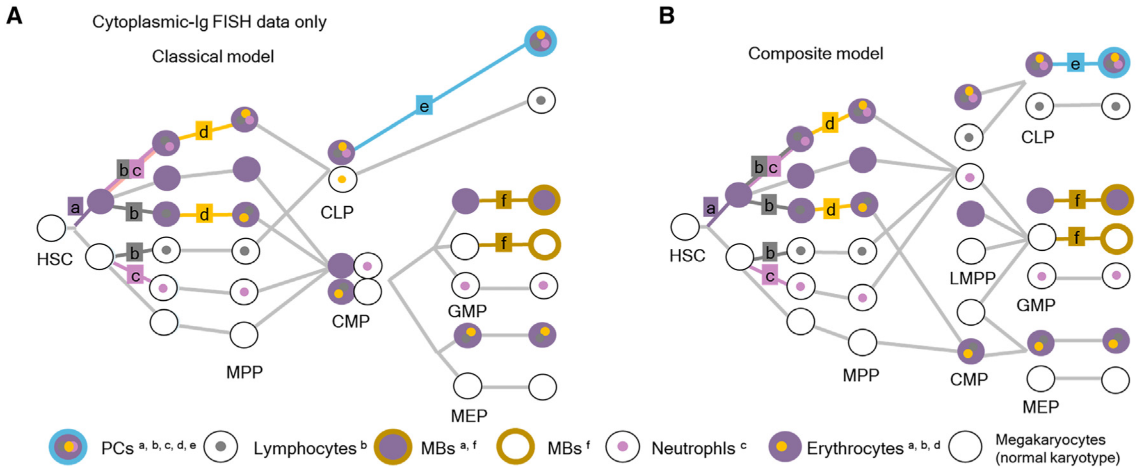


**Figure 3. Hypothetical hematopoietic lineage model inferred from FISH data**

Interphase FISH, cytoplasmic-Ig FISH, and direct bone BM smear FISH data were fit to the classical model (A) and the composite model (B) of hematopoiesis. Genomic changes are labeled using lowercase letters: a, polysomy 19; b, polysomy 15; c, polysomy 21; d, polysomy 11; e, polysomy 9; and f, polysomy 8 and 20q deletion.

Abbreviations: CLP, common lymphoid progenitor; CMP, common myeloid progenitor; GMP, progenitor of granulocytes and macrophages; HSC, hematopoietic stem cell; LMPP, lymphoid-primed multipotent progenitor; MB, myeloblast; MEP, progenitor of megakaryocytes and erythroid cells; MPP, multipotent progenitor; PC, plasma cell.

PCM. *ZNF676*, *PRPF3*, *GPC3* (c.355G>T), *KMT2C*, and *KDR* mutations were detected in the initial diagnosis specimen, whereas *GPC3* (c.352G>T) was identified in the initial and last follow-up specimens (Table 1).

To elucidate the origin and differentiation of PCs and MBs, it is important to accurately separate the two different populations. Using cytogenetic aberration data obtained from cytoplasmic-Ig FISH and direct BM smear FISH, we speculated a hypothetical hematopoietic tree explaining the origin and differentiation of neoplastic PCs and MBs in the context of the classical model of hematopoiesis[10] in our case. We first sought to display genomic changes that were revealed using cytoplasmic-Ig FISH and direct BM smear FISH to compare the results and cross-validate our inferred lineage model (Figures 3 and 4). Genomic changes that occurred in this patient were grouped into 6 groups. The first group, a, had polysomy 19; the second group, b, had polysomy 15; the third group, c, had polysomy 21; the fourth group, d, had polysomy 11; the fifth group, e, had polysomy 9; and the sixth group, f, had polysomy 8 and 20q deletion. We deduced the level of the clonal changes in hierarchy along the hematopoietic tree. Neoplastic PCs had genomic changes of a, b, c, d, and e. Meanwhile, MBs consisted of 2 heterogeneous populations; MB with a and f, and MB with f (Figure 3A). Meanwhile, our data also fit well with the composite model[11] (Figure 3B).

Using data analyzed by Hema-seq WGS, copy-number alterations and single-nucleotide variations were determined. These data not only matched the cytoplasmic-Ig FISH and direct BM smear FISH information but also provided a more comprehensive hypothetical hematopoietic tree. At the copy-number level, the genomic changes that occurred in this patient were divided into nine groups (Figure 4). The first change, A, displayed polysomies 3, 15, and 19. Group B had polysomies 5, 6, 7, 9, 11, 18, and 21 and loss of 11q and TP53. Group C showed a loss of chromosome 2. Group D displayed 4q and X loss. Group E had polysomy 8 and 20q loss, and group F had polysomy 6. Group G exhibited polysomy 16 and a loss of 10. Group H displayed polysomy 21, and group I displayed polysomies 11, 15, and 19 and chromosomal loss in 4. Compared with bulk target sequencing data, we were able to detect three additional groups determined by single-nucleotide variations. Group 1 contained *ZNF676* (c.1564G>T) and *PRPF3* (c.11C>A). Group 2 contained *GPC3* (c.352G>T, c.355G>T), *KMT2C* (c.2576G>T), and *KDR* (c.409T>G). Group 3 patients had *ATM* (c.1262C>A).

Neoplastic PCs (nPCs) consisted of two heterogeneous populations: $nPC_{A,B,C,1}$ and $nPC_{A,B,D,2}$. Meanwhile, MBs consisted of two heterogeneous populations: $MB_{A,E,F,3}$ and $MB_{A,E}$ (Figure 4). The neoplastic cells consisted of four types of heterogeneous populations: cells with A, B, C, and 1; cells with A, B, D, and 2; cells with A, E, and F; and cells with A and E. We deduced the level of clonal changes in the hierarchy along the hematopoietic tree. At the highest hierarchy, change A partially occurred at the level of lymphoid-primed multipotent progenitors (LMPPs); thus, LMPPs are mixed populations: LMPPs with normal karyotype, and LMPPs with A. When LMPPs branch into multilymphoid progenitors (MLPs) and progenitors of granulocytes and macrophages (GMPs), cells with A become MLPs, whereas heterogeneous cells with normal karyotypes and polysomy become GMPs. MLPs then differentiate into PCs with the acquisition of the additional change B. Thereafter, nPCs acquire two different changes: one with additional changes C and 1, and another with changes D and 2. Ultimately, there are two kinds of nPCs: nPCs with changes A, B, C, and 1, and nPCs with changes A, B, D, and 2. Meanwhile, GMPs acquire change E and then separate into two kinds of MB populations: MB with changes A, E, and F, and MB with changes A and E. Additionally, neutrophils and eosinophils displayed unique copy-number alterations

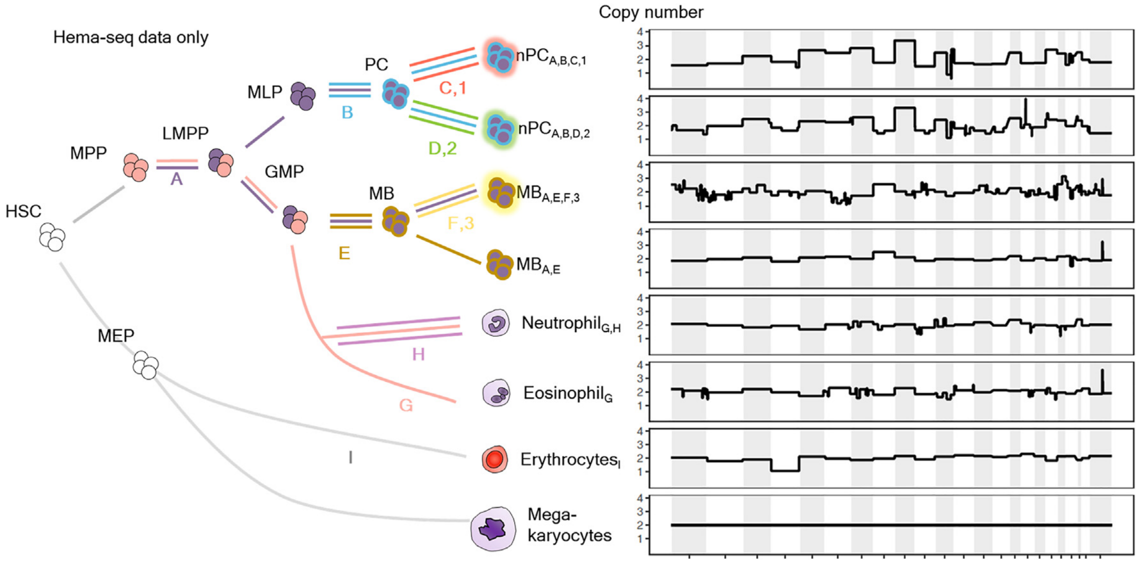


**Figure 4. Hematopoietic was resolved using Hema-seq**

Hematopoietic lineage determined using Hema-seq only. A refers to polysomies 3, 15, and 19; B, polysomies 5, 6, 7, 9, 11, 18, and 21, as well as 11q and *TP53* loss; C, 2 loss; D, 4q loss and X loss; E, polysomy 8 and 20q loss; F, polysomy 6; G, polysomy 16 and 10 loss; H, polysomy 21; and I, polysomies 11, 15, and 19 and 4 loss. 1 refers to *ZNF676* (c.1564G>T) and *PRPF3* (c.11C>A); 2 refers to *GPC3* (c.352G>T, c.355G>T), *KMT2C* (c.2576G>T), and *KDR* (c.409T>G); and 3 refers to *ATM* (c.1262C>A).

(change G plus H and change G, respectively) that seem to have been gained from GMPs. Erythrocytes changed after division from MEPs. Megakaryocytes showed a normal copy number. Recent studies have demonstrated the characteristics of pre-leukemic hematopoietic stem cells (HSCs). Pre-leukemic HSCs harbor common somatic mutations found in myeloid leukemic cells and serve as a potential reservoir for relapse. In the present case, we presumed that clones with polysomy 19 would be pre-myeloma as well as a pre-leukemic progenitor populations.

In addition, the single-nucleotide variations detected in Hema-seq in the PC populations were shown to present changes in the mutation burden in bulk targeted sequencing (Figure 5). As shown by the changes in PCs in Figure 2D, the single-nucleotide variations seemed to diminish after the first treatment cycle of decitabine. As such, the potential of Hema-seq is that whole genomes of each lineage cell that are cataloged differently according to cytoplasmic Ig-FISH or Wright-Giemsa can be analyzed at the single-nucleotide level. The data presented show high accordance with FISH results and bulk targeted sequencing results. An interesting observation was that *ATM* (c.1262C>A) showed a decrease in bulk targeted sequencing after two cycles of decitabine and diminished after decitabine cycles 3–4 with one cycle of lenalidomide and dexamethasone. At the time point when myeloma achieved complete remission, while AML persisted, bulk sequencing still showed persistence of *ATM* (c.1262C>A). In Hema-seq, this variant was only detected in a population of MBs that were thought to persist throughout the treatment. When matched with bulk targeted sequencing, we observed a decrease and eventual elimination of this population after four cycles of decitabine and one cycle of lenalidomide and dexamethasone. Meanwhile, the MB population containing *DDX41* (c.679A>G) and *BAX* (c.280C>T) persisted after 365 days. Collectively, Hema-seq not only delineates populations according to genomic aberrations but also links morphologically defined information and genomic aberration information.

## DISCUSSION

In a rare case of concurrent occurrence of AML and PCM, we identified PC-specific and MB-specific genetic changes using Hema-seq, which showed how each clonal change occurs during the differentiation of hematopoietic cells and revealed the nature of heterogeneous populations. In particular, genomic aberrations, in terms of copy-number alterations and single-nucleotide variations, were observed in heterogeneous populations. Both PCs and MBs originated from a common clone with polysomy 19. We selected PCs by morphology, direct BM smear FISH, and cytoplasmic-Ig FISH, which accurately identifies PCs by recognizing fluorescent cytoplasmic light chains. The results of the present study may influence the selection of tailored treatment regimens and follow-up plans. Moreover, we found evidence of common progenitor PCs and MBs, supporting the classical and composite model of hematopoiesis. We also suggest this as evidence for the presence of pre-myeloma and pre-leukemic clones. From a technical perspective, the scalability of the DLP used in Hema-seq has potential for use in large-scale BM cell analysis, and the ability to obtain genomic information on cytopathologically typed cells present in rare disease will hopefully aid in the development of diagnostics and treatments for patients with rare diseases. Because Hema-seq is compatible with conventional protocols for direct BM smear FISH, cytoplasmic Ig-FISH, and Wright-Giemsa staining without modifications, it is readily adoptable to samples prepared in general laboratory medicine practice. Another key strength comes from the fact

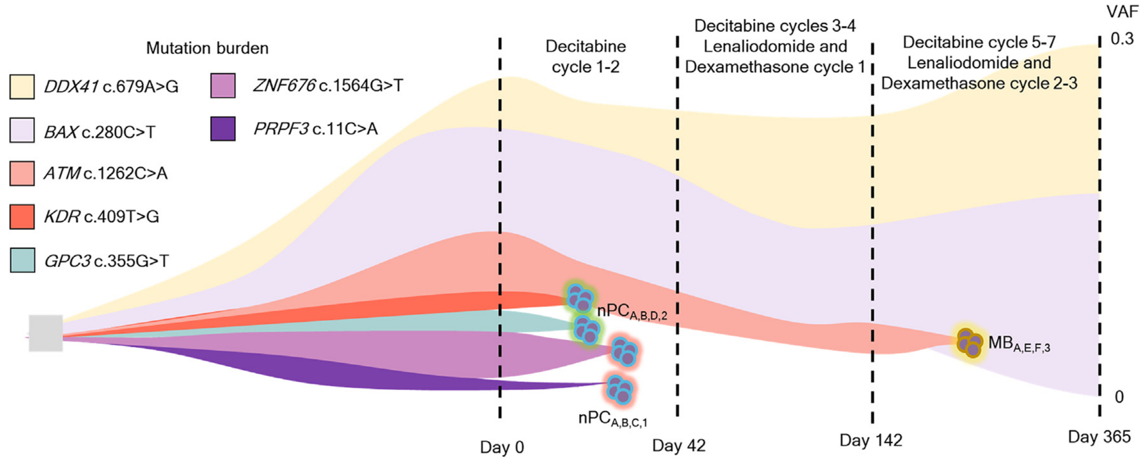


**Figure 5. The treatment effects of different cell types were revealed with Hema-seq and bulk targeted sequencing**
Changes in mutational burden throughout treatment inferred with bulk targeted sequencing data and Hema-seq data. Abbreviations: HSC, hematopoietic stem cell; GMP, progenitors of granulocytes and macrophages; LMPP, lymphoid-primed multipotent progenitor; MB, myeloblast; MLP, multilymphoid progenitor; CMP, multipotent common myeloid progenitor; MEP, megakaryocyte-erythroid progenitor cell.

that Hema-seq is useful in designing retrospective clinical studies, as it is compatible with cells that are prepared for routine/gold-standard laboratory techniques. Therefore, Hema-seq can be used in archived samples just like the sample used in this study. We used SLACS for sorting the cells, but Hema-seq is also compatible with conventional laser capture microdissection systems. Lastly, although the limitation of this study is that the cells were not truly analyzed at single-cell level due to limitations in whole-genome amplification (WGA) technology, if novel WGA technologies such as primary template-directed amplification (PTA)[12] can be applied to stained hematological cells, the resolution of Hema-seq will increase. This strength of Hema-seq will contribute to discovering genomic insights from the archived samples that are not compatible with other technologies.

## Limitations of the study

Our study, while shedding light on a unique case of a rare simultaneous occurrence of hematological malignancies, is rooted in specific contexts that warrant some points of caution. The primary foundation of our analysis relies on a singular, unique case, and broad applicability to diverse hematological malignancies, as well as validation of our findings in additional cohorts, is warranted. The cytogenetic models we propose, derived chiefly from FISH data, are based on certain assumptions, especially surrounding the evolution of clones. Though we consider these models robust, they are not without inherent uncertainties that hypothetical constructions often carry. Additionally, the precision of Hema-seq remains to be rigorously validated in scenarios with rare aberrations or lower tumor burdens. Interphase FISH, a cornerstone of our study, has intrinsic limitations, particularly when not extensively multiplexed, potentially leading to a partial capture of a cell's genomic landscape. Lastly, while every effort has been made to refine Hema-seq, the potential for technical biases, inherent in most sequencing methodologies, may still influence our results. We analyzed 20–30 cell pools for the clinical samples because of the DNA quality of the clinical sample, which is damaged due to staining and fixation process. This approach improves the sensitivity; however, it is important to know that some copy-number variation (CNV)/single-nucleotide variation (SNV) heterogeneity could be hindered for this sensitivity. It is crucial to interpret our findings within these confines, even as they pave the way for deeper explorations in the field.

## STAR★METHODS

Detailed methods are provided in the online version of this paper and include the following:

- KEY RESOURCES TABLE
- RESOURCE AVAILABILITY
  - Lead contact
  - Materials availability
  - Data and code availability
- EXPERIMENTAL MODEL AND STUDY PARTICIPANT DETAILS
  - Patient samples
  - Cell culture
- METHOD DETAILS
  - Flow cytometry
  - G-banding
  - Interphase fluorescent *in situ* hybridization (FISH)
  - Cytoplasmic-immunoglobulin FISH (cytoplasmic-Ig FISH)
  - Direct bone marrow smear FISH
  - SLACS isolation
  - Isolation of each hematopoietic cell
  - Direct library preparation and next generation sequencing (NGS)
  - Multiple displacement amplification
- QUANTIFICATION AND STATISTICAL ANALYSIS

- ○ Copy number analysis
- ○ CV and MAPD calculation of cell line data
- ○ Target sequencing analysis
- ○ Demultiplexing
- ○ Alignment algorithm and analysis tools
- ○ Variant analysis strategy in bulk targeted sequencing
- ○ Variant analysis strategy in Hema-seq samples

## SUPPLEMENTAL INFORMATION

Supplemental information can be found online at https://doi.org/10.1016/j.crmeth.2023.100617.

## ACKNOWLEDGMENTS

This research was supported by the Ministry of Science and ICT (MSIT) of the Republic of Korea and the National Research Foundation of Korea (NRF-2020R1A3B3079653 to S.K. and RS-2023-00266110 to A.C.L.); by the Industrial Strategic Technology Development Program (20024391, Development of a Personalized Breast Cancer Diagnosis and Treatment Technology-Product Using Single-Cell Multi-Omics Analysis and In Situ Sequencing of Circulating Tumor Cells to A.C.L.) funded by the Ministry of Trade, Industry and Energy (MOTIE, Korea); and by the Ministry of Education of the Republic of Korea (2021R1I1A1A01045372 to A.C.L.).

## AUTHOR CONTRIBUTIONS

D.S.L. and S.K. conceived of the idea and supervised the work. D.J., A.C.L., and K.S. developed the Hema-seq method. A.C.L. developed the automated target isolation program. K.S., J.K, S.L., and A.C. developed the Hema-seq bioinformatics pipeline. D.J. and M.H.H. provided and prepared the specimen from the patient. T.R., O.K., and Y.J. developed the reagents used in Hema-seq. S.K. and D.S.L. supervised this work.

## DECLARATION OF INTERESTS

A.C.L., S.L., and S.K. hold shares in Meteor Biotech Co., Ltd.

Received: November 22, 2022
Revised: May 24, 2023
Accepted: September 21, 2023
Published: October 17, 2023

## REFERENCES

1. Schieppati, A., Henter, J.I., Daina, E., and Aperia, A. (2008). Why rare diseases are an important medical and social issue. Lancet *371*, 2039–2041.
2. Dean, F.B., Hosono, S., Fang, L., Wu, X., Faruqi, A.F., Bray-Ward, P., Sun, Z., Zong, Q., Du, Y., Du, J., et al. (2002). Comprehensive human genome amplification using multiple displacement amplification. Proc. Natl. Acad. Sci. USA *99*, 5261–5266.
3. Lu-Qun, W., Hao, L., Xiang-Xin, L., Fang-Lin, L., Ling-Ling, W., Xue-Liang, C., and Ming, H. (2015). A case of simultaneous occurrence of acute myeloid leukemia and multiple myeloma. BMC Cancer *15*, 724–726.
4. Akashi, K., Harada, M., Shibuya, T., Fukagawa, K., Kimura, N., Sagawa, K., Yoshikai, Y., Teshima, T., Kikuchi, M., Niho, Y., et al. (1991). Simultaneous occurrence of myelomonocytic leukemia and multiple myeloma: involvement of common leukemic progenitors and their developmental abnormality of "lineage infidelity". J. Cell. Physiol. *148*, 446–456.
5. Stepanauskas, R., Fergusson, E.A., Brown, J., Poulton, N.J., Tupper, B., Labonté, J.M., Becraft, E.D., Brown, J.M., Pachiadaki, M.G., Povilaitis, T., et al. (2017). Improved genome recovery and integrated cell-size analyses of individual uncultured microbial cells and viral particles. Nat. Commun. *8*, 84.
6. Zong, C., Lu, S., Chapman, A.R., and Xie, X.S. (2012). Genome-wide detection of single-nucleotide and copy-number variations of a single human cell. Science *338*, 1622–1626.
7. Kim, S., Lee, A.C., Lee, H.B., Kim, J., Jung, Y., Ryu, H.S., Lee, Y., Bae, S., Lee, M., Lee, K., et al. (2018). PHLI-seq: constructing and visualizing cancer genomic maps in 3D by phenotype-based high-throughput laser-aided isolation and sequencing. Genome Biol. *19*, 158.
8. Zahn, H., Steif, A., Laks, E., Eirew, P., VanInsberghe, M., Shah, S.P., Aparicio, S., and Hansen, C.L. (2017). Scalable whole-genome single-cell library preparation without preamplification. Nat. Methods *14*, 167–173.
9. Xi, L., Belyaev, A., Spurgeon, S., Wang, X., Gong, H., Aboukhalil, R., and Fekete, R. (2017). New library construction method for single-cell genomes. PLoS One *12*, e0181163.
10. Reya, T., Morrison, S.J., Clarke, M.F., and Weissman, I.L. (2001). Stem cells, cancer, and cancer stem cells. Nature *414*, 105–111.
11. Adolfsson, J., Månsson, R., Buza-Vidas, N., Hultquist, A., Liuba, K., Jensen, C.T., Bryder, D., Yang, L., Borge, O.-J., Thoren, L.A.M., et al. (2005). Identification of Flt3+ lympho-myeloid stem cells lacking erythro-megakaryocytic potential: a revised road map for adult blood lineage commitment. Cell *121*, 295–306.
12. Gonzalez-Pena, V., Natarajan, S., Xia, Y., Klein, D., Carter, R., Pang, Y., Shaner, B., Annu, K., Putnam, D., Chen, W., et al. (2021). Accurate genomic variant detection in single cells with primary template-directed amplification. Proc. Natl. Acad. Sci. USA *118*, e2024176118.
13. Jeong, D., Lee, D.S., Kim, N., Choi, S., Kim, K., Kim, S.M., Im, K., Park, H.S., Yun, J., Lim, K.M., et al. (2019). Prevalence of germline predisposition gene mutations in pediatric acute myeloid leukemia: Genetic background of pediatric AML. Leuk. Res. *85*, 106210.

## STAR★METHODS

### KEY RESOURCES TABLE

| REAGENT or RESOURCE | SOURCE | IDENTIFIER |
|---|---|---|
| Antibodies | | |
| Polyclonal rabbit anti-human kappa light chains/FITC | Dako Denmark A/S, Glostrup, Denmark | Cat#F0198; Lot#20010443; RRID:AB_2335707 |
| Polyclonal rabbit anti-human lambda light chains/FITC | Dako Denmark A/S, Glostrup, Denmark | Cat#F0199; Lot#20002483; RRID:AB_2335708 |
| Chemicals, peptides, and recombinant proteins | | |
| pTXB1 cloning vector | Addgene, Massachusetts, USA | N/A |
| REPLI-g Single Cell Kit | Qiagen, Hilden, Germany | 150343 |
| Genomiphi V2 DNA amplification kit | Cytiva, Massachusetts, USA | 25-6600-31 |
| Proteinase K from Tritirachium album | Sigma Aldrich, St. Louis, Missouri, USA | 39450-01-6 |
| SYTO™ 13 Green Fluorescent Nucleic Acid Stain | Invitrogen, Massachusetts, USA | S7575 |
| TG Nextera® XT Index Kit v2 Set A (96 Indices, 384 Samples) | Illumina, California, USA | TG-131-2001 |
| KAPA HiFi HotStart Library Amp Kit | Roche, Basel, Switzerland | N/A |
| LSI RUNX1/RUNX1T1 | Vysis, Downers Grove, IL, USA | Cat#08L70-020; Lot#475956 |
| LSI PML/RARA | Vysis, Downers Grove, IL, USA | Cat#01N36-020; Lot#479451 |
| LSI CBFB | Vysis, Downers Grove, IL, USA | Cat#05N44-020; Lot#472359 |
| LSI IGH/MAF | Vysis, Downers Grove, IL, USA | Cat#05N32-020; Lot#476755 |
| LSI IGH/FGFR3 | Vysis, Downers Grove, IL, USA | Cat#01N69-020; Lot#475715 |
| LSI CEP8 | Vysis, Downers Grove, IL, USA | Cat#30–170008; Lot#475235 |
| LSI ETV6/RUNX1 | Vysis, Downers Grove, IL, USA | Cat#08L66-020; Lot#475613 |
| LSI CDKN2A | MetaSystems, Altlussheim, Germany | Cat#D-5053-100-OG; Lot#19042 |
| LSI CDKN2C/CKS1B | MetaSystems, Altlussheim, Germany | Cat#D-5049-100-OG; Lot#18216 |
| LSI MLL | MetaSystems, Altlussheim, Germany | Cat#D-5060-100-OG; Lot#18451 |
| LSI DLEU/LAMP | MetaSystems, Altlussheim, Germany | Cat#D-5054-100-OG; Lot#19193 |
| LSI IGH | MetaSystems, Altlussheim, Germany | Cat#D-5061-100-OG; Lot#19053 |
| LSI 20q12 | MetaSystems, Altlussheim, Germany | Cat#D-5020-100-OG; Lot#19401 |
| LSI TP53/CEP17 | MetaSystems, Altlussheim, Germany | Cat#D-5103-100-OG; Lot#19401 |
| LSI TCF3 | CytoCell, Cambridge, UK | Cat#LPH019; Lot# 161114-011 |
| Critical commercial assays | | |
| Celemag beads | Celemics, Inc., Seoul, Republic of Korea | N/A |
| Next Generation Sequencing | ATG Lifetech, Inc., Seoul, Republic of Korea | N/A |
| Target capture sequencing | Celemics, Inc., Seoul, Republic of Korea | N/A |
| Deposited data | | |
| Hema-seq data | This paper | PRJNA1010154 |
| Experimental models: Cell lines | | |
| SK-BR-3 cell line | ATCC, Virginia, USA | HTB-30™ |
| Software and algorithms | | |
| Custom Scripts | This paper | https://doi.org/10.5281/zenodo.8325422 |

### RESOURCE AVAILABILITY

#### Lead contact

Further information and requests for resources and reagents should be directed to and will be fulfilled by the lead contact, Sunghoon Kwon (skwon@snu.ac.kr).

### Materials availability

This study did not generate new unique reagents.

### Data and code availability

- Hema-seq data have been deposited at NCBI Sequence Read Archive under the accession number PRJNA1010154 and is publicly available as of the date of publication.
- Custom scripts to reproduce the analyses reported in this study are available on GitHub [https://github.com/BiNEL-SNU/Hema-seq]. An archival DOI is provided in the key resources table.
- Any additional information required to reanalyze the data reported in this paper is available from the lead contact upon request.

## EXPERIMENTAL MODEL AND STUDY PARTICIPANT DETAILS

### Patient samples

In this study the sample was obtained from 69-year-old Korean woman which showed simultaneous occurrence of acute myeloid leukemia and plasma cell myeloma. This study was approved by the Institutional Review Board (IRB) of Seoul National University Hospital (IRB No. 1911-158-1082). This study was performed in accordance with the Declaration of Helsinki. The requirement for informed consent was waived by the IRB of Seoul National University Hospital due to the retrospective nature of this study.

### Cell culture

The SK-BR-3 cell line was used to determine copy number alterations using whole-genome sequencing. The cell line was purchased from the American Type Culture Collection (ATCC) and cultured according to the manufacturer's instructions.

## METHOD DETAILS

### Flow cytometry

The used antibody reagent information is as follows: anti-CD45-APC (10 μL; Beckman Coulter, USA), anti-CD33-PE (20 μL; Beckman Coulter) anti-CD34-PC5 (20 μL; Beckman Coulter), anti-CD2-FITC (20 μL; Beckman Coulter), anti-CD10-PE (20 μL; Beckman Coulter), anti-CD3-PC5 (10 μL; Beckman Coulter), anti-CD56-FITC (20 μL; Becton Dickinson, USA), anti-CD117-PE (20 μL; Beckman Coulter), anti-CD41-PC5 (10 μL; Beckman Coulter), anti-CD13-FITC (20 μL; Beckman Coulter), anti-CD19-PE (20 μL; Beckman Coulter), anti-CD7-PC5 (10 μL; Beckman Coulter), anti-CD20-FITC (20 μL; Beckman Coulter), anti-CD5-PE (20 μL; Beckman Coulter), anti-TdT-FITC (20 μL; Beckman Coulter), anti-cytoplasmic CD79a-PE (20 μL; Beckman Coulter), anti-cytoplasmic CD3-PC5 (10 μL; Beckman Coulter), anti-cytoplasmic IgM-PE (10 μL; SouthernBiotech, USA), anti-cytoplasmic CD22-PC5 (10 μL; Beckman Coulter) and anti-MPO-FITC (20 μL; Beckman Coulter).

Four types of antibody reagents with different fluorescence were used per tube, and each antibody was mixed with 100 μL of BM aspirate. After 15 min, red blood cell lysis was performed using 2mL of VersaLyse Lysing Solution (Beckman coulter). The tubes were centrifuged at 3000 rpm for 1 min and washed using IsoFlow Sheath Fluid (Beckman coulter). For cytoplasmic markers, fixation and permeabilization reagents (IntraPrep Permeabilization Reagent, Beckman coulter) were additionally used. Beckman Coulter Navios flow cytometer (Beckman Coulter) and Kaluza (Beckman Coulter) software were used for flow cytometric test and data analysis, respectively.

### G-banding

Buffy coat isolated from heparinized BM samples was cultured in RPMI 1640 media (Gibco, ThermoFisher Scientific, New York, USA) supplemented with fetal bovine serum (Gibco), Antibiotic-Antimycotic and L-Glutamine (200 mM) (Gibco) with 35 μL of Interleukin-4 (STEMCELL Technologies, USA) at 37°C in 5% CO2 for 3 days. After centrifugation at 1200 rpm for 8 min and washing, cell pellet was moved to 10 mL of pre-made RPMI 1640 supplement media and cultured with 35 μL of interleukin-4 at 37°C for 2 days. A total of 100 μL of Colcemid (Gibco) was added and the specimen was cultured for 50 min. After centrifugation, Potassium chloride (Sigma-Aldrich, Germany) was added at 37°C for 20 min. Following fixation with 1 mL of Carnoy's solution, Leishman's G-banding stain was performed according to the standard protocol. Metafer4 (MetaSystems, Germany) was used for karyogram analysis.

### Interphase fluorescent *in situ* hybridization (FISH)

Interphase FISH analysis was performed on mononuclear cells from bone marrow (BM) aspirates to detect cytogenetic abnormalities related to acute myeloid leukemia and plasma cell myeloma. We used LSI RUNX1/RUNX1T1 (Vysis, Downers Grove, IL, USA), LSI PML/RARA (Vysis), LSI CBFB (Vysis), LSI IGH/MAF (Vysis), LSI IGH/FGFR3 (Vysis), XL CDKN2A (MetaSystems, Altlussheim, Germany), XL CDKN2C/CKS1B (MetaSystems), XL MLL (MetaSystems), XL DLEU/LAMP (MetaSystem), XL IGH (MetaSystems) and LSI TP53/17cen (MetaSystem). Considering the results of G-banding, we additionally added CEP8 (Vysis), XL Del(20q) (Metasystems), TCF3 (CytoCell, Cambridge, UK), and LSI ETV6/RUNX1 (Vysis). The detailed experimental procedures have been previously documented.[13]

### Cytoplasmic-immunoglobulin FISH (cytoplasmic-Ig FISH)

Cytoplasmic Ig FISH staining was performed on slides used for FISH. After removing the cover glass, the slides were washed five times with phosphate buffered saline (PBS) and dried. A total of 5 μL of polyclonal rabbit anti-human kappa and lambda light chain antibodies (Dako Denmark A/S, Glostrup, Denmark), which were labeled with FITC, were applied to the slides. The slides were incubated in a wet chamber for 30–60 min, washed with PBS twice for 3–5 min each, and completely dried. Counterstaining was performed using diamidino-2-phenylindole (DAPI II) (Vysis). Then, interphase FISH was performed as described above. At least 20 interphase plasma cells were analyzed.

### Direct bone marrow smear FISH

A BM aspiration slide was stained with Wright-Giemsa stain. Images of the distribution of neutrophils, erythrocytes, lymphocytes, and megakaryocytes were obtained prior to the procedure. The slide was de-stained twice with 100% methanol (MeOH) for 5 min each at room temperature. After air-drying, the slides were soaked in 4% paraformaldehyde for 20 min at room temperature. Using 4× SSC, the slides were washed at room temperature using 4 × SSC. Following these procedures, FISH was performed as previously described.[13]

### SLACS isolation

Spatially resolved laser-activated cell sorting (SLACS) was used to isolate cells of interest. After the cells were stained with FISH, cytoplasmic-Ig FISH, or Wright-Giemsa, SLACS automatically sorted 20–30 cells in a PCR tube cap filled with proteinase K and nuclease-free water. Each PCR tube cap was incubated for an hour at 50°C and proteinase K was denatured with chymostatin. The reactions then underwent direct library preparation as described below.

### Isolation of each hematopoietic cell

Cell isolation was performed based on a combination of cytoplasmic Ig-FISH, interphase FISH, direct BM smear FISH and W-G staining. Plasma cells with polysomy 11 and plasma cells with polysomy 19 were sorted based on cytoplasmic Ig-FISH while myeloblasts with 20q deletion and myeloblasts with polysomy 19 were isolated based on interphase FISH. Moreover, neutrophils and eosinophils were distinguished by their nucleus shape (e.g., band-shaped nucleus of neutrophils) and autofluorescence on FISH-stained slide, respectively. Meanwhile, erythrocytes and megakaryocytes were isolated using Wright-Giemsa stained slide. In addition, direct BM smear FISH was performed for neutrophils, eosinophils, megakaryocytes and lymphocytes, which are more clearly distinguishable by Wright-Giemsa stain rather than FISH stained slide.

### Direct library preparation and next generation sequencing (NGS)

Hyperactive *E54K* and *L372P* mutations were introduced into wild-type Tn5 using a pTXB1 cloning vector (Addgene). pTXB1 Tn5 and its mutant were expressed and purified according to a protocol described in the literature. Using Tn5 proteins, lysed samples were incubated for 20 min for tagmentation at 65°C. Tagmentation was stopped using proteinase K, which was denatured for 20 min at 65°C after incubating for 1 h at 37°C. The tagged genome fragments were subjected to polymerase chain reaction (PCR) for 13 cycles. The second PCR was performed for 7 cycles. The amplicons were purified using Celemag beads (Celemics Inc., Republic of Korea). Paired-end sequencing (150 bp) was performed using an Illumina NextSeq sequencing platform.

### Multiple displacement amplification

For the process of cell lysis and the denaturation of lysed gDNA, a mixture was created using 1 μL of the template containing either gDNA or cells, 3 μL of cell lysis solution (400 mM KOH, 10 mM EDTA, 100 mM DTT), 2 μL of PBS (REPLI-g Single Cell Kit, Qiagen), and 1 μL of 500 μM random hexamer. The mixture was subjected to cell lysis and denaturation on ice for a duration of 20 min. Following this, 3 μL of a neutralization buffer composed of 400 mM HCl and 600 mM Tris-HCl (pH 7.5) was added to counteract the lysis buffer.

Subsequently, an MDA master mix of 40 μL was added, containing 23 μL of water, 5 μL of 10× phi29 DNA polymerase reaction buffer [500 mM Tris-HCl, 100 mM MgCl2, 100 mM (NH4)2SO4, 40 mM DTT, pH 7.5], 4 μL of 25 mM dNTP, 2 μL of 1 mM random hexamer, 2 μL of phi29 DNA polymerase (Genomiphi V2 DNA amplification kit, Cytiva, cat. no. 25-6600-31), 3.2 μL of 40% (w/v) PEG 8000, 0.25 μL of 1 M DTT, 0.5 μL of 50 μM SYTO 13 Green Fluorescent Nucleic Acid Stain (Invitrogen), and 0.05 μL of 500 nM ROX. The 50 μL MDA reaction mix was incubated at 30°C for 12 h, followed by inactivation at 65°C for 10 min. The addition of SYTO 13 and ROX fluorescent dyes allowed for real-time monitoring of MDA amplification. The first 3 h of the MDA reaction were observed in real-time (RT-MDA), and the Applied Biosystems 7500 Fast Real-Time PCR System was employed for quantitative monitoring.

## QUANTIFICATION AND STATISTICAL ANALYSIS

### Copy number analysis

Copy number alterations (CNAs) were estimated based on NGS read density using the variable binning method. Briefly, the human genome is divided into 10,000 variable-sized bins (median bin size = 276 kbp), and bin size is known to have an equal expected number of uniquely mapped reads. The read depth of each bin was first normalised based on the GC content of each bin. The GC-normalised

read density was divided by the median read depth to convert read depth into chromosomal copy number scale, assuming near-diploidy of the samples. Circular binary segmentation (CBS) is then used to detect copy number alteration events. Finally, MergeLevels was used to merge the detected segments that showed insignificant changes to suppress false-positive CNA detection. The average estimated copy number for each bin in one segment is regarded as the true copy number of the segment.

### CV and MAPD calculation of cell line data

We performed CNA calling using carious bin numbers. To assess the uniformity of each bin, we calculated the coefficient of variation (CV) and median absolute pairwise difference (MAPD).

CV was calculated by

$$CV = \frac{\text{stdev of readcount per bin}}{\textit{Mean of readcount per bin}}$$

Additionally, to calculate the MAPD, we first scaled the pairwise differences scaled by mean(d). Then Compute the meadian absolute deviation of d, defined as

$$\text{Median}(|\text{d} - \text{Median}(\text{d})|)$$

### Target sequencing analysis

Capture probes were designed and chemically synthesised to hybridise with the target region. Genomic DNA was sheared and processed for Illumina sequencing using the following steps: end repair, dA-tailing, adapter ligation, and pre-PCR for the indexed NGS library. NGS-prepared gDNA and capture probes were hybridised to capture the target regions using a Celemics Target Enrichment Kit. The captured regions were then further amplified by post-PCR to enrich the sample amount. The captured library was then sequenced using an Illumina NextSeq550 instrument (Illumina, San Diego, CA, USA) using 2 × 150 bp.

### Demultiplexing

BCL2FASTQ version 2.19.1.403 (Illumina) was used to demultiplex base-call image files into individual sequence read files (FASTQ format). All options and parameters followed default settings.

### Alignment algorithm and analysis tools

Sequencing adapters were removed using ‘AdapterRemoval’ version 2.2.2. after low-quality bases (BQ0 and N sequences) were removed using the native Python code. All sequencing reads were aligned to the hg19 human genome using Burrows-Wheeler Aligner (BWA-MEM) software. The program uses the Burrows-Wheeler transform algorithm to index the human genome sequence to calculate the constant complexity of each sequencing read. Post-align and recalibration processes were performed using ‘Picard’ version 1.115 (http://broadinstitute.github.io/picard) and ‘GATK’ version 4.0.4.0. We performed variant calling using the GATK haplotype caller. All detailed parameters and options followed the best practices.

### Variant analysis strategy in bulk targeted sequencing

Synonymous variants, variants with >1% minor allele frequency (MAF) and non-coding region mutations were excluded. For somatic mutation analysis, variants with alternative depth <10, VAF <5%, or total depth <250 were excluded. Exceptions were allowed if at least one of the common gene variants had a depth $\geq$10, VAF $\geq$5%, or total depth $\geq$250. Consequently, the three somatic mutations were rescued.

### Variant analysis strategy in Hema-seq samples

For the single nucleotide variants in direct library preparation samples, we first searched all reads that were aligned to the variant in the target sequencing data. Then, we determined the presence of a variant sequence in the reads. Thus, we profiled the variants in each cell. For validation, we used DLP data from the SK-BR-3 cell line. We extracted gDNA from the cell line and termed this a single nucleotide variant using whole exome sequencing data. We also observed whether that specific variant was present in our cell line sequencing data.

Cell Reports Methods, Volume *3*

## Supplemental information

# Hema-seq reveals genomic aberrations in a rare simultaneous occurrence of hematological malignancies

**Dajeong Jeong, Amos C. Lee, Kyoungseob Shin, Jinhyun Kim, Myoung Hee Ham, Changhee Lee, Sumin Lee, Ahyoun Choi, Taehoon Ryu, Okju Kim, Yushin Jung, Sunghoon Kwon, and Dong Soon Lee**

Supplementary figures

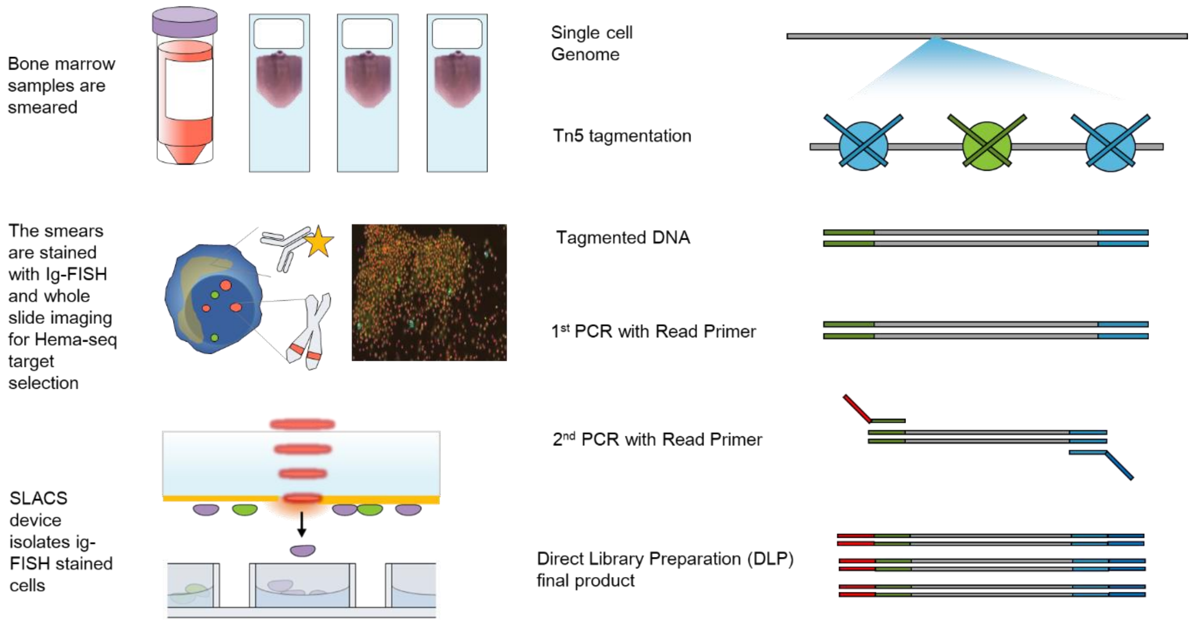


**Figure S1. Hema-seq isolates cytoplasmic-Ig FISH stained cells with SLACS and Direct Library Preparation is performed to amplify genome, related to Figure 1.**

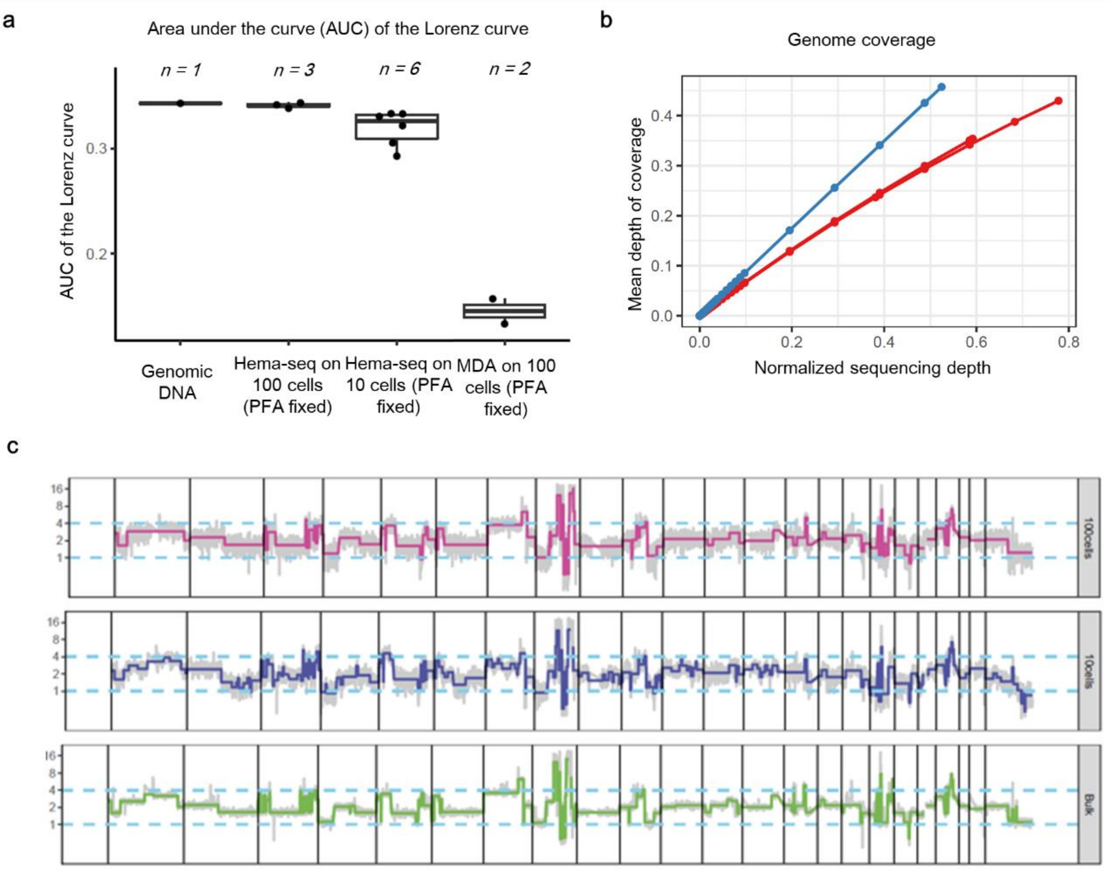


**Figure S2: Hema-seq is validated using the cell line, related to Figure 1.** (**a**) Area under the curve of the Lorenz curve for different methodologies for low-input whole genome sequencing and cell numbers. MDA is an abbreviation for multiple displacement amplification, which is the most widely used whole genome amplification method. (**b**) Genome coverage of the Hema-seq (red) compared to bulk (blue). (**c**) Copy number analysis for comparison between Hema-seq prepared samples (violet and blue) and bulk population (green).

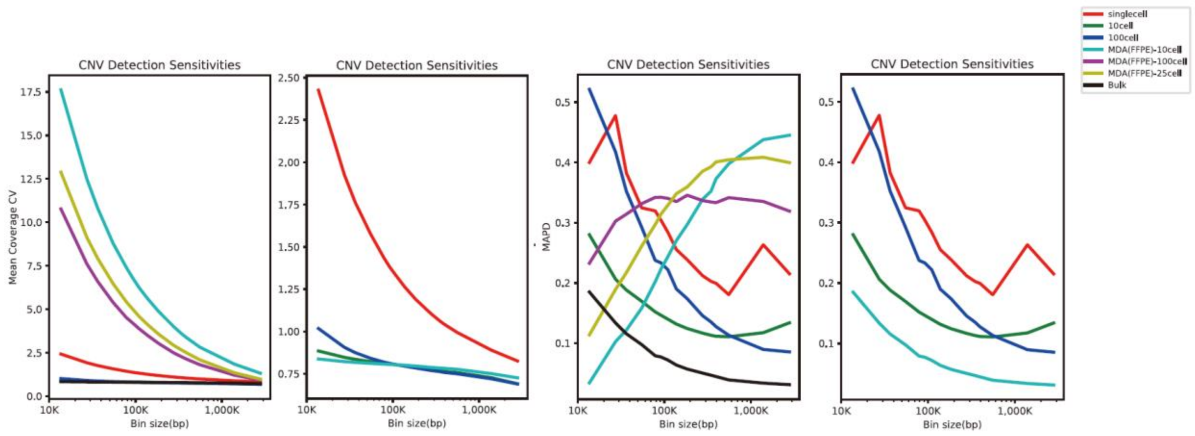


**Figure S3: CNV detection sensitivities of Hema-seq is compared with MDA protocol by mean coverage CV & MAPD of increasing bin size, related to Figure 1.** Hema-seq protocol is applied to single cell(n=9), 10 cells(n=6), 100 cells(n=3) and for comparison MDA protocol is applied to 10 cells(n=2), 25 cells(n=2), 100 cells(n=1) which is performed at FFPE sample. Reference is the bulk sequencing data.

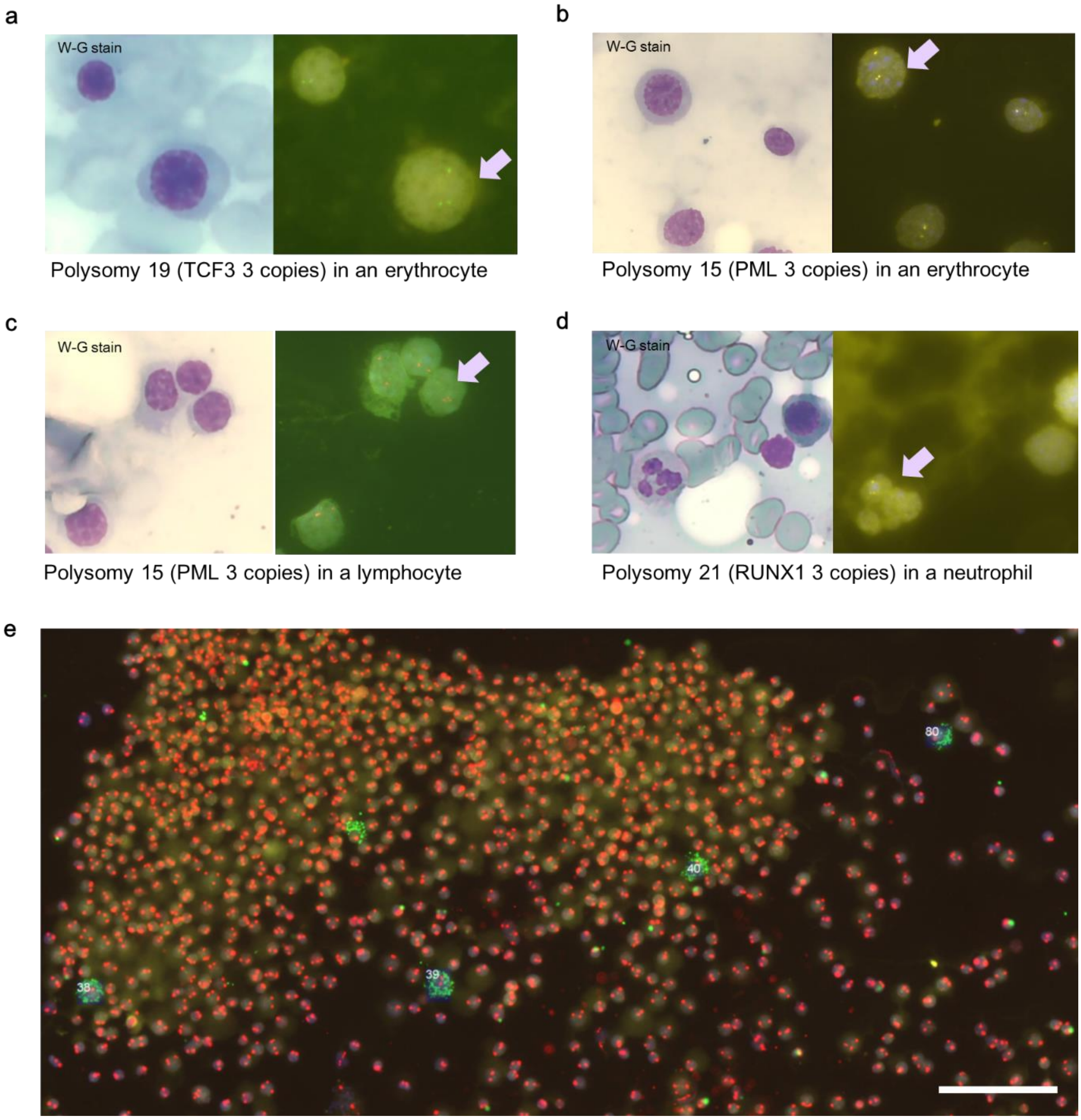


**Figure S4. Wright-Giemsa staining, direct BM smear FISH and cytoplasmic-Ig FISH results of the patient analysed related to Figure 2.** Wright-Giemsa (W-G) staining and direct BM smear FISH results show polysomy 19 (TCF3 3 copies) in an erythrocyte (**a**), polysomy 15 (PML 3 copies) in an erythrocyte (**b**), polysomy 15 (PML 3 copies) in a lymphocyte (**c**), and polysomy 21 (RUNX1 3 copies) in a neutrophil (**d**). (**e**) Cytoplasmic Ig-FISH results and markings for Hema-seq for selective isolation of different cell types. Scale bar, 100 μm

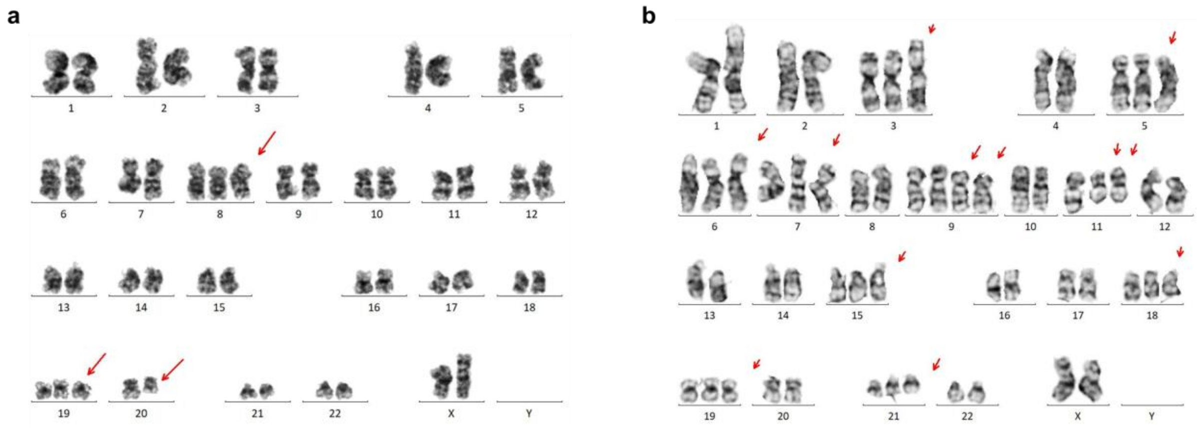


**Figure S5. Karyograms of the analysed person is used for initial diagnosis related to Figure 2**. Clone 1 (48,XX, +8,+19,del(20q)) representing leukemia clone (a) and clone 2 (57,XX, +3, +5,+6,+7,+9,+9,+11,+15,+18,+19,+21) suggestive of plasma cell myeloma clone (b), which were confirmed by interphase FISH and cytoplasmic-Ig FISH. Cytogenetic changes are labelled using red arrows.

**Supplementary Table**

**Table S1.** Target sequencing panel is used to find the single nucleotide variant in the clone, related to Figure 4, Figure 5, Table 1.

| |
|---|
| *ABCA7, ABCB7, ABCC1, ABL1, ABRAXAS1, ACD, ACKR1, ACTB, ACVR2B, ACYP2, ADA, AK2, AKAP13, AKAP9, AKT1, AKTIP, ALK, ALMS1, ALPK2, AMER1, ANKRD24, ANKRD26, AP3B1, APC, ARHGAP26, ARID1A, ARID1B, ARID2, ARID3A, ARID4B, ASXL1, ASXL2, ASXL3, ATM, ATR, ATRX, B2M, BAP1, BARD1, BAX, BCAS3, BCL2L1, BCL10, BCL11B, BCL2, BCL2L11, BCL6, BCL7A, BCL9, BCOR, BCORL1, BCR, BICD1, BIRC3, BLM, BLNK, BLOC1S6, BMPR1A, BRAF, BRCA1, BRCA2, BRCC3, BRD2, BRD4, BRD7, BRINP3, BRIP1, BRPF1, BTG1, BTG2, BTG3, BTK, BTLA, BUB1, BUB1B, CACNA1E, CALR, CARD11, CARD6, CASP10, CASP8, CBL, CBLB, CBLC, CBX5, CBX7, CCDC80, CCND1, CCND3, CD200, CD27, CD36, CD3D, CD3E, CD40LG, CD58, CD70, CD79A, CD79B, CDC73, CDH1, CDH23, CDK12, CDK4, CDKN1B, CDKN1C, CDKN2A, CDKN2B, CDKN3, CEBPA, CELSR2, CEP164, CHD1, CHD2, CHD8, CHEK2, CHRNA3, CHRNA5, CHRNB4, CIAO1, CIAO2B, CIAO3, CIC, CIITA, CLPTM1L, CMYA5, CNOT1, COIL, COL4A2, COL6A3, CPNE3, CREBBP, CRLF2, CSF1R, CSF3R, CSTF2T, CTC1, CTCF, CTNNB1, CTSS, CUL9, CUX1, CXCL12, CXCR4, CYLD, DAP3, DAXX, DCAF4, DCLK1, DCLRE1B, DCLRE1C, DDB2, DDX1, DDX11, DDX23, DDX3X, DDX41, DDX54, DHX29, DHX32, DHX58, DICER1, DIS3, DIS3L2, DKC1, DKK2, DLEU1, DLEU2, DMD, DNAH9, DNM2, DNMT1, DNMT3A, DNMT3B, DST, DYRK4, EBF1, ECT2L, EED, EEF1E1, EGFR, EGR2, ELANE, EP300, EPCAM, EPHA2, EPHA3, EPHA7, ERBB2, ERCC2, ERCC3, ERCC4, ERCC5, ERCC6, ETS1, ETV3, ETV6, EXT1, EXT2, EZH2, FAH, FANCA, FANCB, FANCC, FANCD2, FANCE, FANCF, FANCG, FANCI, FANCL, FANCM, FAS, FASLG, FAT4, FBXO11, FBXW7, FGFR2, FGFR3, FH, FLCN, FLG, FLT3, FOXL2, FOXN1, FOXO1, FOXP3, FUBP1, FYB1, G6PC3, G6PD, GAR1, GATA1, GATA2, GATA3, GEMIN2, GFI1, GLI1, GNA11, GNA13, GNAQ, GNAS, GNB1, GPC3, GPC4, GSTP1, HAX1, HCK, HDAC2, HDAC3, HDAC7, HEATR1, HIST1H1E, HNF1A, HNRNPK, HMBOX1, HOXB13, HRAS, HUWE1, IDH1, IDH2, IKZF1, IKZF2, IKZF3, IL2RG, IL7R, IRAK1, IRAK4, IRF1, IRF4, IRF8, ITK, ITPKB, JAK1, JAK2, JAK3, JMJD1C, KDM2B, KDM3B, KDM4C, KDM5C, KDM6A, KDM6B, KDR, KIAA0355, KIF20B, KIT, KLF6, KLHL6, KLK3, KMT2A, KMT2B, KMT2C, KMT2D, KRAS, L2HGDH, LAMB4, LAMTOR2, LEF1, LIG4, LMO2, LRP1B, LRRK1, LSP1, LUC7L2, LYST, MAD1L1, MAGT1, MALT1, MAP2K1, MAP2K2, MAP2K4, MAP3K1, MAP3K14, MAP4K1, MAPK1, MAX, MDM2, MED12, MEF2B, MEF2C, MEN1, MET, METTL3, MIR155, MKI67, MLH1, MMS19, MPDZ, MPHOSPH6, MPL, MRE11, MSH2, MSH6, MSMB, MST1R, MTA2, MTOR, MUC16, MUTYH, MXRA5, MYB, MYC, MYCN, MYD88, MYLK2, MYO3A, NAF1, NBN, NCF4, NF1, NF2, NFE2L2, NFKB2, NFKBIA, NFKBIE, NHEJ1, NHP2, NKX2-1, NOP10, NOTCH1, NOTCH2, NOTCH3, NPM1, NR3C1, NRAS, NRK, NSD1, NTRK1, NUP214, OBSCN, OR6K3, ORAI1, P2RY8, PALB2, PALLD, PARD3, PARP1, PARP2, PASD1, PASK, PAX5, PBRM1, PCLO, PDGFC, PDGFRA, PDGFRB, PDS5B, PDSS2, PHF6, PHLPP1, PHOX2B, PIF1, PIGA, PIGT, PIK3C3, PIK3CA, PIK3R1, PIM1, PINX1, PKD1L2, PLCG2, PLEKHG5, PLRG1, PML, PMS1, PMS2, PNP, POLG, POLH, POLR2A, POSTN, POT1, POU2F2, PPP2R1A, PRDM1, PRDM16, PRDM9, PRF1, PRKAR1A, PRKCG, PRKD3, PRKDC, PRPF3, PRPF40B, PRPF8, PRSS1, PTCH1, PTEN, PTPN11, PTPN14, PTPRC, PTPRT, PWWP3A, RAB27A, RAC2, RAD21, RAD50, RAD51, RAD51AP1, RAD51B, RAD51C, RAD51D, RAD54L, RAF1, RAG1, RAG2, RAPGEF1, RARA, RB1, RBBP4, RBMX,* |

| |
|---|
| *RECQL, RECQL4, RECQL5, REL, RELN, RET, RFTN1, RFX7, RFXAP, RING1, RIPK1, RIT1, RMRP, RNF213, RPL11, RPL19, RPL27, RPL35A, RPL5, RPN1, RPS10, RPS14, RPS17, RPS19, RPS24, RPS26, RPS27, RPS6KA6, RPS7, RTEL1, RUNX1, SAMD9, SAMD9L, SAMHD1, SAP130, SBDS, SCML2, SCRIB, SDHA, SDHAF2, SDHB, SDHC, SDHD, SENP6, SETBP1, SETD2, SF1, SF3A1, SF3B1, SGK1, SH2B3, SH2D1A, SHOC2, STK11, SLC37A4, SLC7A7, SLITRK6, SLX4, SMAD1, SMAD4, SMAD7, SMARCA2, SMARCA4, SMARCB1, SMARCD2, SMARCE1, SMC1A, SMC3, SMC5, SMG1, SMO, SNRNP200, SOCS1, SOS1, SPEN, SPINK1, SRP72, SRRM2, SRSF2, SRSF6, SRSF8, STAG2, STAT3, STAT5B, STAT6, STIM1, STK4, STK11, STK32A, STK33, STK36, STN1, STRIP2, STX11, STXBP2, SUDS3, SUFU, SUMO2, SUPT5H, SUZ12, SYK, SYNE1, TAF1, TAL1, TAZ, TBL1XR1, TBX1, TCF12, TCF4, TEN1, TENT4B, TENT5C, TEP1, TERC, TERF1, TERF2, TERF2IP, TERT, TET1, TET2, TGFB2, TGM6, TGM7, THPO, THRB, TINF2, TMEM127, TMEM30A, TNFAIP3, TNFRSF14, TNFSF9, TNKS, TNKS1BP1, TNKS2, TOX3, TP53, TRA2B, TRAF3, TRIM37, TRIO, TSC1, TSC2, TSHR, TTBK1, TTC27, TTN, TYK2, TYW1, U2AF1, U2AF1L4, U2AF2, UBA3, UBE2A, UGGT1, ULK4, UNC13B, UNC13D, UNC5C, UNC5D, USB1, VHL, VPS13A, VPS13B, VPS45, WAC, WAPAL, WAS, WEE1, WIPF1, WNK3, WNK4, WRAP53, WRN, WT1, XIAP, XPA, XPC, XPO1, XRCC2, XRCC6, ZAP70, ZBTB33, ZBTB7B, ZFHX3, ZMYM3, ZNF208, ZNF311, ZNF608, ZNF676, ZNF708, ZRSR2* |

**Table S2**. Results of direct BM smear FISH on neutrophil, erythrocytes, lymphocytes, neutrophil and megakaryocytes, related to Figure 2.

| Direct BM smear FISH | Neutrophil | Erythrocyte | Lymphocyte | Megakaryocytes |
|---|---|---|---|---|
| Polysomy 19 (TCF3 3 copies) | 0/13 (0.0%) | 4/68 (5.9%) | 0/11 (0.0%) | N/A |
| Polysomy 8 (CEP8 3 copies) | 0/7 (0.0%) | 0/188 (0.0%) | 0/52 (0.0%) | 0/0 (0.0%) |
| Polysomy 11 (MLL 3 copies) | 0/6 (0.0%) | 1/17 (5.9%) | 0/16 (0.0%) | N/A |
| Polysomy 15 (PML 3 copies) | 0/38 (0.0%) | 5/209 (2.4%) | 4/113 (3.5%) | 0/0 (0.0%) |
| Polysomy 21 (RUNX1 3 copies) | 1/3 (33.3%) | 0/5 (0.0%) | 0/10 (0.0%) | 0/0 (0.0%) |
